# Genome-Wide Identification and Functional Characterization of the Carboxylesterase (CXE) Gene Family in Melon (*Cucumis melo* L.): *CmCXE7* as a Cold-Responsive Regulator of Ester Metabolism

**DOI:** 10.3390/plants15203144

**Published:** 2026-10-14

**Authors:** Yongjie Xia, Jie Liu, Huijun Zhang, Zhuoran Huang, Xiuxiu Zhu, Hu Li

**Affiliations:** 1 College of Life Science, Huaibei Normal University, Huaibei 235000, China; 18805601148@163.com (Y.X.); whjiejiel@163.com (J.L.); zhhuijun@126.com (H.Z.); hzrds@hotmail.com (Z.H.); 20231504037@chnu.edu.cn (X.Z.); 2 Anhui Biological Breeding Engineering Research Center for Watermelon and Melon, Huaibei Normal University, Huaibei 235000, China

**Keywords:** melon, carboxylesterase, gene family, genome-wide identification, functional characterization, *CmCXE7*, metabolomics, fruit ripening

## Abstract

Melon fruit aroma depends on the balance between ester biosynthesis and hydrolysis, yet the carboxylesterase (CXE) family responsible for hydrolysis remains uncharacterized in this crop. Here, a genome-wide identification of the CXE gene family in melon revealed 22 *CmCXE* genes. Phylogenetic analysis revealed that Group III members cluster with functionally validated ester-hydrolyzing CXEs from peach, tomato, and strawberry, and their expression peaks during the climacteric and post-climacteric stages—the same window when melon aroma volatiles accumulate. We further functionally characterized *CmCXE7*, a cold-responsive member, through subcellular localization and heterologous overexpression in tomato. *CmCXE7* localized predominantly to the plasma membrane with a weak nuclear signal. Non-targeted metabolomics revealed 776 differentially accumulated metabolites (DAMs) based on VIP > 1 and *p* < 0.05, with 11 of 14 high-magnitude ester-related changes being downregulated. These results identify Group III genes as candidate regulators of melon ester metabolism and identify *CmCXE7* as a cold-responsive CXE candidate that may participate in ester metabolism. The potential relevance of *CmCXE7* to fruit ripening and postharvest storage warrants further investigation, providing a candidate gene for future functional studies and molecular breeding of melon aroma quality.

## 1. Introduction

Fruit aroma, defined by the composition and dynamic turnover of volatile organic compounds (VOCs), is a central determinant of fruit flavor quality and consumer preference [1,2,3]. Among the diverse classes of VOCs, volatile esters constitute the largest and most functionally important group, contributing the characteristic fruity and floral notes in a wide range of fruits, including apple, strawberry, banana, melon, and tomato [4,5]. The biosynthesis and catabolism of volatile esters are tightly regulated during fruit ripening and postharvest storage, with cultivar genotype, maturity stage, and environmental conditions collectively shaping the final aroma profile [6,7,8].

The metabolic balance of volatile esters is governed by two opposing enzymatic activities: alcohol acyltransferases (AATs) catalyze the esterification of alcohols with acyl-CoA donors to form esters, whereas carboxylesterases (CXEs) mediate the reverse hydrolysis reaction, releasing the corresponding alcohol and carboxylate [9,10]. CXEs belong to the α/β hydrolase superfamily and are structurally characterized by a conserved GXSXG catalytic motif and an HGGG oxyanion hole, which together form the catalytic triad essential for ester hydrolysis [11,12,13]. Functional studies have confirmed the role of CXE genes in fruit aroma regulation across multiple species. In tomato, *SlCXE1* was identified as a key regulator of flavor volatile variation, with high expression in ripe fruit leading to rapid hydrolysis of acetate esters [14]. In peach, *PpCXE1* was shown to hydrolyze volatile esters during ripening, and stable overexpression in tomato significantly reduced ester content in vivo [15]. In strawberry, *FanCXE1* contributes to ester catabolism during ripening by converting esters to alcohols; transient RNAi suppression resulted in increased levels of major volatile esters and decreased alcohols [16]. More recently, genome-wide analyses in pineapple identified 20 *AcCXE* genes, with *AcCXE4* and *AcCXE7* showing the strongest association with aroma formation during fruit ripening [17]. Beyond fleshy fruits, CXEs have also been implicated in stress responses, hormone signaling, and secondary metabolism in Arabidopsis, rice, cotton, and soybean, highlighting the functional diversification of this gene family across plant lineages [11,18,19]. In particular, jasmonate (JA) signaling plays a central role in coordinating plant defense and stress responses [20], suggesting that hormone crosstalk may contribute to the transcriptional regulation of CXE genes.

Genome-wide identification and bioinformatic characterization have become standard approaches for systematically exploring gene families in plants [21]. Such analyses typically integrate phylogenetic classification, chromosomal localization, gene structure, conserved motif architecture, cis-regulatory element profiling, and expression pattern analysis, providing a comprehensive framework for hypothesis generation and candidate gene prioritization [22,23]. This strategy has been successfully applied to the CXE family in several species, including peach [24], rice [18], cotton [11], and pineapple [17], revealing lineage-specific expansion patterns and candidate genes for functional validation.

Melon (*Cucumis melo* L.) is an economically important horticultural crop cultivated worldwide, prized for its distinctive and diverse aromatic profiles [25]. Volatile esters, particularly acetate esters, are the dominant contributors to melon fruit aroma, and their biosynthesis is closely linked to fatty acid and amino acid metabolic pathways [26,27]. Recent studies have shown that postharvest chilling significantly reduces melon flavor by suppressing volatile acetate ester biosynthesis, and transcriptomic analysis has revealed that several melon CXE homologs are upregulated under cold storage, suggesting a potential role for CXEs in aroma loss during chilling injury [28,29]. Plasma membrane–localized receptor-like kinases, particularly the CrRLK1L family, have emerged as key sensors of stress-induced membrane changes and mediators of abiotic stress signaling in crops [30], providing a potential mechanistic link between cold perception and the transcriptional regulation of aroma-related genes such as *CXE7*. However, despite these advances, a systematic, genome-wide identification and functional characterization of the CXE gene family in melon remains lacking. The evolutionary relationships, structural features, expression patterns, and potential functions of most melon CXE members are still largely unknown.

Building on preliminary work in our laboratory using the ‘Huaitian No. 1’ melon cultivar, 14 aroma-related candidate genes were previously identified through transcriptomic analysis under low-temperature storage conditions. Among these, *CmCXE7* and several other CXE family members exhibited expression patterns closely associated with aroma changes during cold storage, implicating the CXE family as a key regulatory module in melon aroma dynamics. Nevertheless, the genome-wide landscape of the melon CXE family, the phylogenetic context of these candidate genes, and the mechanistic basis underlying their expression regulation remain to be elucidated.

In the present study, we conducted a comprehensive genome-wide identification and bioinformatic analysis of the CXE gene family in melon, covering phylogenetic relationships, syntenic characteristics, gene structure, conserved motifs, cis-acting elements, physicochemical properties, expression patterns across tissues and developmental stages, and protein structural modeling. We further investigated the function of the candidate gene *CmCXE7* through heterologous expression in tomato and a wide-ranging metabolomic analysis, providing preliminary evidence for its potential role in ester metabolism. This integrated study aims to reveal the potential functions of CXE family members in melon fruit aroma formation. We particularly focus on *CmCXE7*, a cold-responsive member whose overexpression is associated with reduced ester accumulation, and discuss its potential relevance to fruit ripening and postharvest storage.

## 2. Materials and Methods

### 2.1. Identification of Melon CmCXE Genes and Construction of the Phylogenetic Tree

The reference genome sequences of melon (*Cucumis melo* L., cv. DHL92, v4.0) [31] and cucumber (*Cucumis sativus* L., cv. ChineseLong, v2.0) were obtained from the Cucurbit Genomics Database v2 (CuGenDBv2, http://cucurbitgenomics.org/v2/) [32]. To identify CXE gene family members in melon, a previously reported melon *CXE* gene (identified in a prior study from our laboratory) was used as the entry point in the Ensembl Plants database (https://plants.ensembl.org/index.html) [33]. The paralogue prediction function of Ensembl Plants was then employed to retrieve all putative melon CXE paralogues. For comparative evolutionary analysis within the Cucurbitaceae family, cucumber CXE orthologues were also retrieved from Ensembl Plants using the same approach. A total of 22 candidate *CmCXE* and 26 candidate *CsCXE* genes were obtained. All candidate sequences were subsequently validated for the presence of the conserved α/β hydrolase fold domain using the NCBI Conserved Domain Database (CDD, https://www.ncbi.nlm.nih.gov/Structure/cdd/wrpsb.cgi, accessed on 17 November 2025), and candidates lacking the characteristic esterase domain or with incomplete functional annotations were removed after manual curation. The confirmed genes were systematically named *CmCXE1*-*CmCXE22* and *CsCXE1*-*CsCXE26* according to their physical order on the respective chromosomes.

For phylogenetic analysis, full-length amino acid sequences of the 22 CmCXE and 26 CsCXE proteins were aligned using the built-in multiple alignment function in MEGA 11 (accessed on 11 October 2026) [34]. A maximum likelihood (ML) phylogenetic tree was constructed in MEGA 11 with 1000 bootstrap replicates to evaluate branch support.

### 2.2. Syntenic Analysis of Melon CmCXE Genes

For syntenic analysis, genome annotation files for melon (DHL92, v4.0) and cucumber (ChineseLong, v2.0) were obtained from the Cucurbit Genomics Database v2 (CuGenDBv2, http://cucurbitgenomics.org/v2/). The strawberry (*Fragaria vesca*) genome and annotation files were downloaded from the Genome Database for Rosaceae (GDR, https://www.rosaceae.org/) [35], and sequences for previously reported strawberry *CXE* genes (*FvCXE*) [9,36] were retrieved using their accession numbers. Syntenic analysis between melon and cucumber, as well as between melon and strawberry, was performed using the MCScanX function integrated in TBtools software (v1.098769, accessed on 13 October 2025) [37] to identify syntenic blocks and syntenic orthologous *CXE* gene pairs. The phylogenetic tree was constructed as described in Section 2.1 and visualized using the Interactive Tree of Life (iTOL v6, https://itol.embl.de/) [38].

### 2.3. Analysis of Gene Structure, Conserved Motifs, and Promoter Elements of Melon CmCXE Genes

Gene structure analysis was performed using the Gene Structure Display Server (GSDS 2.0, http://gsds.gao-lab.org/) [39] based on the GFF annotation files of melon *CXE* genes retrieved from CuGenDBv2 to visualize the exon–intron organization. For conserved domain analysis, the full-length protein sequences of all 22 CmCXEs were submitted to the NCBI Conserved Domain Database (CDD, https://www.ncbi.nlm.nih.gov/Structure/cdd/wrpsb.cgi, accessed on 18 October 2025) [40,41,42], and the domain architecture was visualized using TBtools (v1.098769) [37]. Conserved motif analysis was performed using the MEME Suite (v5.5.4, https://meme-suite.org/meme/, accessed on 18 October 2025) [43,44], with the maximum number of motifs set to 15, motif width ranging from 6 to 50 amino acids, and site distribution set to zero or one occurrence per sequence. To further validate the conservation of the catalytic core, the full-length protein sequences of the 22 CmCXE genes were aligned using ClustalW integrated in MEGA 11, and the alignment was visualized and shaded using GeneDoc (v2.7; http://www.nrbsc.org/gfx/genedoc/, accessed on 27 August 2026). Conserved and similar residues are indicated by black and gray shading, respectively. The motif distribution patterns were illustrated using TBtools (v1.098769). For cis-acting element analysis, the 2000 bp upstream sequences from the translation start codon of each *CmCXE* gene were extracted as putative promoter regions and submitted to the PlantCARE database (http://bioinformatics.psb.ugent.be/webtools/plantcare/html/, accessed on 19 October 2025) [45] for prediction and functional annotation. The 2000 bp window was selected because most plant cis-regulatory elements cluster within 1–2 kb upstream of the translation start site [46]. Prediction was performed on the positive strand, and redundant elements (identical sequence at the same position) were merged and counted once. In addition to the promoter-focused analysis, full-length gene sequences were also submitted to PlantCARE to capture potential regulatory elements located in introns, UTRs, or downstream regions, providing a complementary global perspective beyond the proximal promoter.

### 2.4. Physicochemical Properties, Protein Structure, and Subcellular Localization Prediction of CmCXEs

The protein sequences encoded by the melon CXE genes were retrieved from the Ensembl Plants database. Physicochemical properties, including amino acid composition, molecular weight, theoretical isoelectric point (pI), instability index, and grand average of hydropathicity (GRAVY), were analyzed using the ExPASy ProtParam online tool (https://web.expasy.org/protparam/, accessed on 19 October 2025) [47]. For three-dimensional protein structure prediction, the full-length amino acid sequences of all 22 CmCXEs were submitted to the AlphaFold2 modeling pipeline [48,49] as implemented on the SWISS-MODEL server (https://swissmodel.expasy.org/, accessed on 20 October 2025) [50,51], with default parameters. The prediction confidence is indicated by the pLDDT (predicted local distance difference test) score. Subcellular localization was predicted for all melon CXE family members using the WoLF PSORT online tool (https://wolfpsort.hgc.jp/, accessed on 21 February 2026) [52]. Transmembrane domains were predicted using TMHMM 2.0 (accessed on 29 August 2026) [53], and signal peptides were predicted using SignalP 6.0 with default parameters [54]; both tools are available from the DTU Health Tech server (https://www.healthtech.dtu.dk/).

### 2.5. Transcriptomic Expression Analysis and Protein–Protein Interaction Network Analysis of the Carboxylesterase Family

Building on the bioinformatic characterization described above, transcriptomic expression analysis was performed to link sequence-based predictions with functional evidence. To comprehensively investigate the expression patterns of melon *CmCXE* genes, publicly available transcriptomic data (fragments per kilobase of transcript per million mapped reads, FPKM) were retrieved from the CuGenDBv2 database (http://cucurbitgenomics.org/v2/expression, accessed on 23 October 2025) by submitting *CmCXE* gene IDs against the melon reference genome (DHL92 v4.0). FPKM expression values were obtained from three independent datasets: (i) tissue-specific profiles in root, stem, leaf, pistillate flower, staminate flower, and ovary (PRJNA803327); (ii) fruit mesocarp developmental stages, including growing, ripening, climacteric, and post-climacteric stages (PRJNA543288); and (iii) four fruit developmental time points at 8, 16, 24, and 32 days after pollination (PRJNA300582). All raw sequencing data are deposited in the NCBI Sequence Read Archive (SRA). Given the broad dynamic range of transcript abundance across the CmCXE gene family (spanning from near-zero to several hundred FPKM), all expression values were log_2_(FPKM + 1)-transformed to stabilize variance, mitigate the dominant influence of highly expressed genes, and render the data suitable for comparative clustering and heatmap visualization. For heatmap presentation, expression values were additionally row-normalized within each gene (scaled relative to the maximum expression of that gene across all samples) to visualize relative expression patterns; this preserves relative patterns but obscures absolute expression differences. The unnormalized raw FPKM values for all three datasets are provided in Table S5. The three datasets were derived from two melon cultivars: PRJNA803327 and PRJNA543288 from cv. Hetao, and PRJNA300582 from cv. Gotgam (*Cucumis melo* var. *makuwa*). Heatmaps were constructed using TBtools (v1.098769).

To further explore the potential biological regulatory networks involving *CmCXE* genes, protein–protein interaction (PPI) analysis was performed using the STRING database (v12.0, https://string-db.org/, accessed on 27 October 2025) [55]. Because a melon-specific protein interaction database is not available in STRING, the carboxylesterase family (COG0657) was used as the query to retrieve a conserved functional interaction module, and a PPI network was constructed with a high confidence threshold of 0.9. The resulting network should be interpreted as a predicted functional module based on conserved orthologous interactions, rather than a melon-specific CmCXE interactome.

### 2.6. Protein Subcellular Localization

Among the 22 identified CmCXE genes, *CmCXE7* was selected for functional investigation based on our prior transcriptomic study, which identified *CmCXE7* as significantly upregulated during low-temperature storage of postharvest melon fruit (see Supplementary materials). To determine its subcellular localization, *Agrobacterium*-mediated transient transformation assays were performed following a protocol established by our research group. The coding sequence of *CmCXE7* was cloned into the pRI101 vector downstream of the GFP coding region, generating a 35S promoter-driven *CmCXE7*-GFP fusion construct. The recombinant plasmid was transformed into *Agrobacterium tumefaciens* strain GV3101 using the freeze–thaw method. Transformed cells were plated on LB agar supplemented with 50 μg/mL kanamycin and 25 μg/mL rifampicin, and incubated at 28 °C for 2 days. Single colonies were cultured overnight in liquid LB medium with the appropriate antibiotics. After centrifugation, bacterial pellets were resuspended in induction medium and incubated statically for 4 h at room temperature, followed by another centrifugation and resuspension in infiltration medium to an OD_600_ of approximately 0.5. The bacterial suspension was infiltrated into the abaxial epidermis of leaves from 4-week-old *Nicotiana benthamiana* plants. At 48 h post-infiltration, the infiltrated leaf areas were excised and observed under a laser scanning confocal microscope (Zeiss LSM 880, Carl Zeiss, Oberkochen, Germany). GFP fluorescence signals were captured and merged with differential interference contrast (DIC) images to determine the subcellular localization.

### 2.7. Non-Targeted Metabolomic Analysis of Transgenic Tomato Fruits

To investigate the potential function of *CmCXE7* in ester metabolism, the full-length coding sequence of *CmCXE7* was cloned into the plant overexpression vector under the control of the CaMV 35S promoter, and the construct was introduced into tomato (*Solanum lycopersicum* cv. MicroTom) via *Agrobacterium tumefaciens*-mediated transformation. From the positive transgenic lines obtained, the transgenic tomato line overexpressing *CmCXE7* (OE4) with high transgene expression was selected and propagated to the T3 generation. Wild-type (WT) plants grown under the same conditions served as the control. For metabolomic analysis, ripe fruits at the red-ripe stage were harvested from both OE4 and WT plants; the pericarp was separated from the placental tissue and immediately frozen in liquid nitrogen, with five biological replicates per group.

Metabolite extraction was performed as follows: approximately 50 mg of frozen pericarp tissue was ground into powder and extracted with 600 μL of methanol containing 2-amino-3-(2-chlorophenyl)-propionic acid (4 ppm) as an internal standard. After vortexing for 30 s, the samples were homogenized with steel balls in a tissue grinder for 60 s at 55 Hz, followed by ultrasonication at room temperature for 15 min. The extracts were then centrifuged at 12,000 rpm and 4 °C for 10 min, and the supernatants were filtered through 0.22 μm membranes for LC-MS analysis.

Non-targeted metabolomic profiling was performed on a Vanquish UHPLC system (Thermo Fisher Scientific, Waltham, MA, USA) coupled with an Orbitrap Exploris 120 mass spectrometer (Thermo Fisher Scientific, USA). Chromatographic separation was achieved on an ACQUITY UPLC HSS T3 column (2.1 × 100 mm, 1.8 μm; Waters, Milford, MA, USA) maintained at 40 °C, with a flow rate of 0.3 mL/min and an injection volume of 2 μL. For positive electrospray ionization (ESI+), the mobile phases consisted of 0.1% formic acid in water (A) and 0.1% formic acid in acetonitrile (B), with the following gradient: 0–1 min, 8% B; 1–8 min, 8–98% B; 8–10 min, 98% B; 10–10.1 min, 98–8% B; 10.1–12 min, 8% B. For negative ESI (ESI-), the mobile phases were 5 mM ammonium formate in water (A) and acetonitrile (B), with the same gradient program. Mass spectrometric detection was operated in both ESI+ and ESI- modes with the following parameters: sheath gas pressure, 40 arb; aux gas flow, 10 arb; spray voltage, 3.50 kV (ESI+) and −2.50 kV (ESI-); capillary temperature, 325 °C; MS1 scan range, *m*/*z* 100–1000; MS1 resolving power, 60,000 FWHM; MS/MS resolving power, 15,000 FWHM; normalized collision energy, 30%.

Raw data were converted to mzXML format using MSConvert (ProteoWizard v3.0.8789, accessed on 21 August 2025) and processed with the R package XCMS (v3.12.0; R v4.2, accessed on 21 August 2025) for feature detection, retention time correction, and alignment (ppm = 15, peakwidth = c (5, 30), mzdiff = 0.01, method = centWave). Metabolite identification was based on accurate mass and MS/MS spectra matched against public databases including HMDB, MassBank, KEGG, LipidMaps, and mzCloud. Multivariate statistical analyses, including principal component analysis (PCA), partial least squares discriminant analysis (PLS-DA), and orthogonal PLS-DA (OPLS-DA), were performed using the R ropls package (v1.22.0, accessed on 21 August 2025). Differential metabolites were screened based on the criteria of variable importance in projection (VIP) > 1 and *p* < 0.05. Pathway enrichment analysis was conducted using MetaboAnalyst (accessed on 21 August 2025), and the identified differential metabolites were mapped to KEGG pathways for biological interpretation.

Quality control was performed using mixed QC samples, internal standards for retention-time calibration, missing-value imputation, and peak-area normalization. Differential accumulation was determined using VIP > 1 and *p* < 0.05, with Benjamini–Hochberg false discovery rate (FDR) correction applied post hoc.

All transgenic experiments were conducted in accordance with the institutional guidelines for genetically modified organism research at Huaibei Normal University.

## 3. Results

### 3.1. Identification and Characterization of the Melon CmCXE Gene Family

A total of 22 CmCXE genes were identified in the melon genome and systematically named *CmCXE1* to *CmCXE22* according to their chromosomal locations (Table S1; motif statistics in Table S3).

Physicochemical property analysis (Table 1) revealed substantial variation among the deduced CmCXE proteins: amino acid length ranged from 253 (*CmCXE3*) to 479 (*CmCXE17*), molecular weight from 28.58 to 53.23 kDa, and theoretical pI from 4.99 to 9.42, with 18 of 22 members (81.8%) being acidic (pI < 7.0). The majority of CmCXEs were predicted as unstable proteins (instability index > 40), and all exhibited negative GRAVY values, indicating hydrophilicity.

Subcellular localization prediction (Table 1) showed that CmCXE members were distributed across the cytoplasm (10 members), chloroplasts (7), nucleus (2), mitochondria (2), and cytoskeleton (1). TMHMM analysis identified four members (*CmCXE9*, *CmCXE10*, *CmCXE17*, *CmCXE19*) with transmembrane helices, whereas SignalP predicted no signal peptides in any CmCXE, suggesting that all family members are non-secretory proteins.

**Figure 1:**
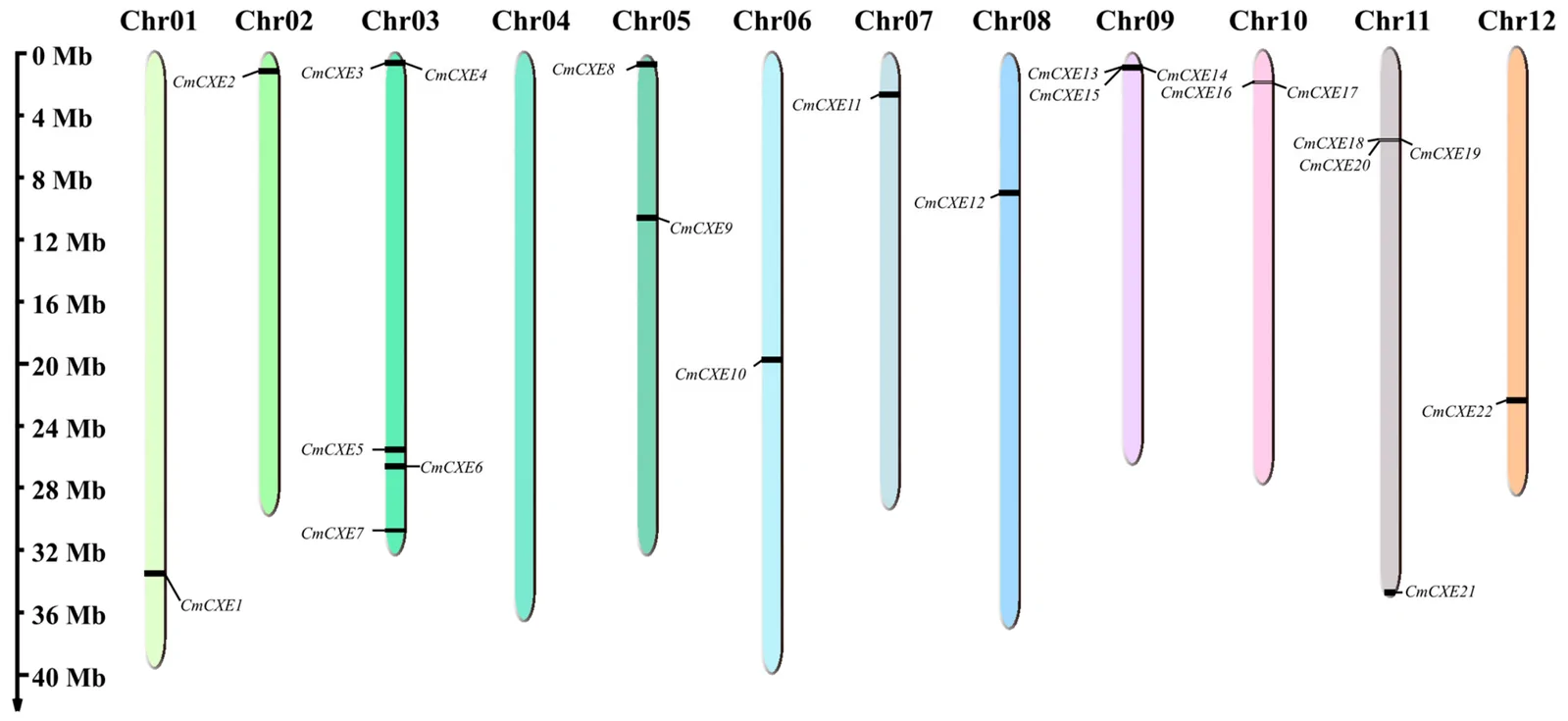
Chromosomal distribution of CmCXE genes in melon. Different colors represent different melon chromosomes. Chromosome numbers are indicated above each chromosome, and CmCXE gene names are shown on both sides.

Chromosomal distribution analysis (Figure 1) revealed that the 22 CmCXE genes were unevenly distributed across 11 of the 12 melon chromosomes, with chromosome 4 harboring none. Chromosome 3 contained the most members (5), followed by chromosomes 11 (4) and 9 (3); the remaining chromosomes harbored one or two genes each. A subset of CmCXE genes were located in subtelomeric regions, a pattern potentially associated with transcriptional regulation or gene duplication events.

### 3.2. Phylogenetic and Syntenic Relationships of the Melon CmCXE Gene Family

**Figure 2:**
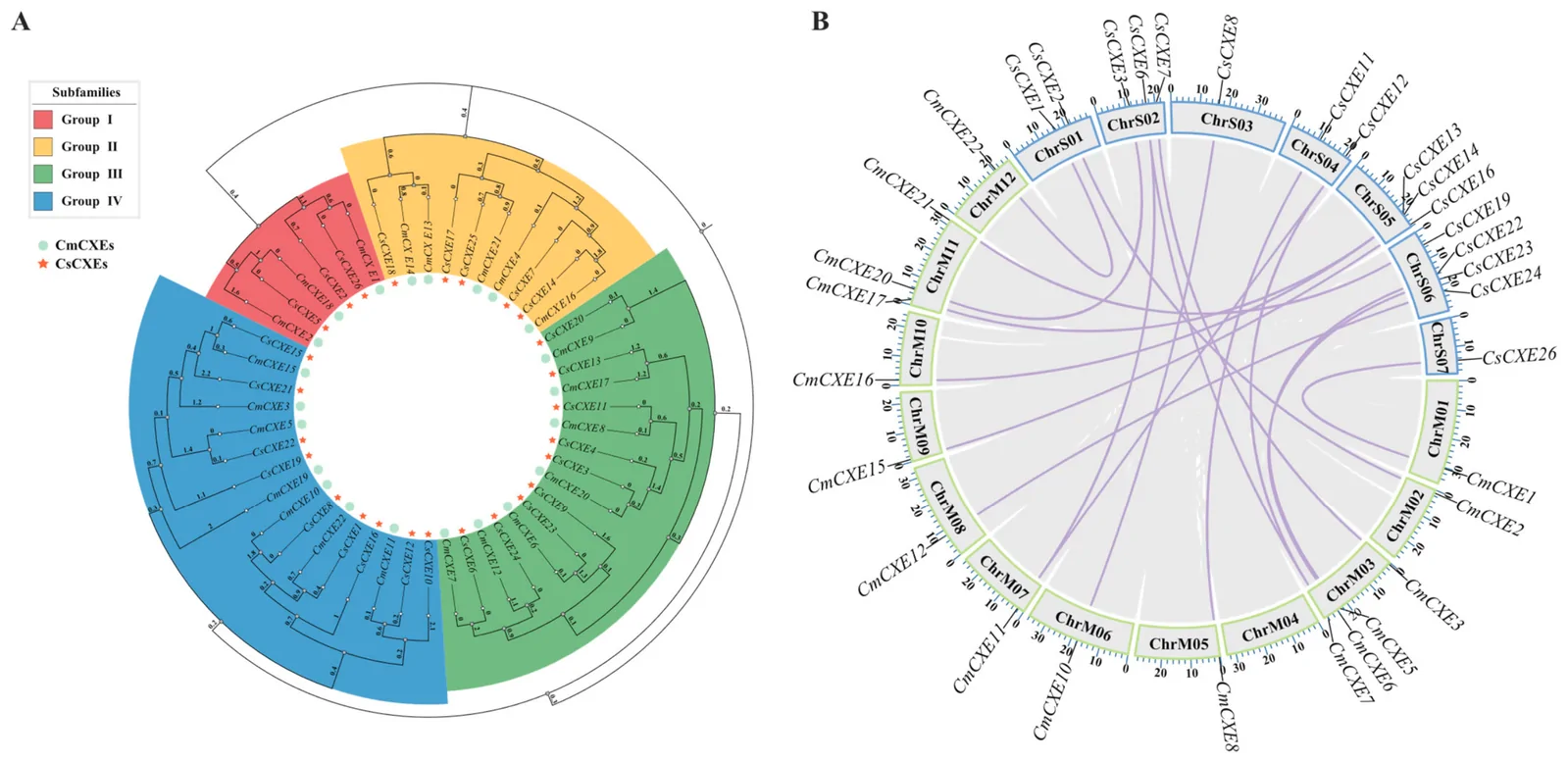
Phylogenetic and syntenic analysis of the melon CmCXE and cucumber CsCXE gene families. (**A**) Circular phylogenetic tree of CXE proteins from melon (22 members) and cucumber (26 members). The tree was constructed using the maximum likelihood method with 1000 bootstrap replicates; bootstrap values ≥ 50% are shown at nodes. Gray squares represent leaf nodes (individual CXE genes), and white stars represent internal nodes (branching points). Branch lengths indicate evolutionary distances; longer branches represent greater genetic divergence. Distinct background colors indicate the four major clades (Groups I–IV). (**B**) Interspecific syntenic analysis between melon and cucumber (Cucurbitaceae). Purple lines highlight orthologous syntenic pairs involving CmCXE genes.

Building on the chromosomal distribution, we next examined the evolutionary relationships of the CXE family across cucurbit crops. To investigate the evolutionary relationships of the CmCXE gene family, 26 cucumber CsCXE genes were identified from Ensembl Plants and renamed according to chromosomal location (Table S1). A maximum-likelihood phylogenetic tree was constructed based on the full-length protein sequences of the 22 CmCXE and 26 CsCXE proteins (Figure 2A; unrooted tree in Figure S1). The 48 CXE proteins clustered into four major clades (Groups I–IV). Groups III and IV were the largest clades, and their members generally exhibited longer branch lengths than those in Groups I and II, suggesting higher evolutionary rates and greater sequence divergence.

Interspecific syntenic analysis between melon and cucumber identified 16 orthologous syntenic pairs, involving 16 CmCXE and 16 CsCXE genes (Figure 2B), indicating high evolutionary conservation of the CXE family within Cucurbitaceae. Cross-species syntenic analysis between melon and strawberry identified 11 syntenic pairs (Figure S2). However, the two functionally validated strawberry CXE genes (*FvCXE2* and *FvCXE3*) [9] were not among these pairs, which is expected given the deep evolutionary divergence between Cucurbitaceae and Rosaceae (~100 million years ago) [56] and indicates that synteny has limited resolution for functional inference at this taxonomic level. Candidate gene prioritization was therefore based primarily on phylogenetic placement (Group III) and expression patterns rather than synteny with validated strawberry genes.

### 3.3. Gene Structure, Protein Domain, and Conserved Motif Analysis of the Melon CmCXE Gene Family

**Figure 3:**
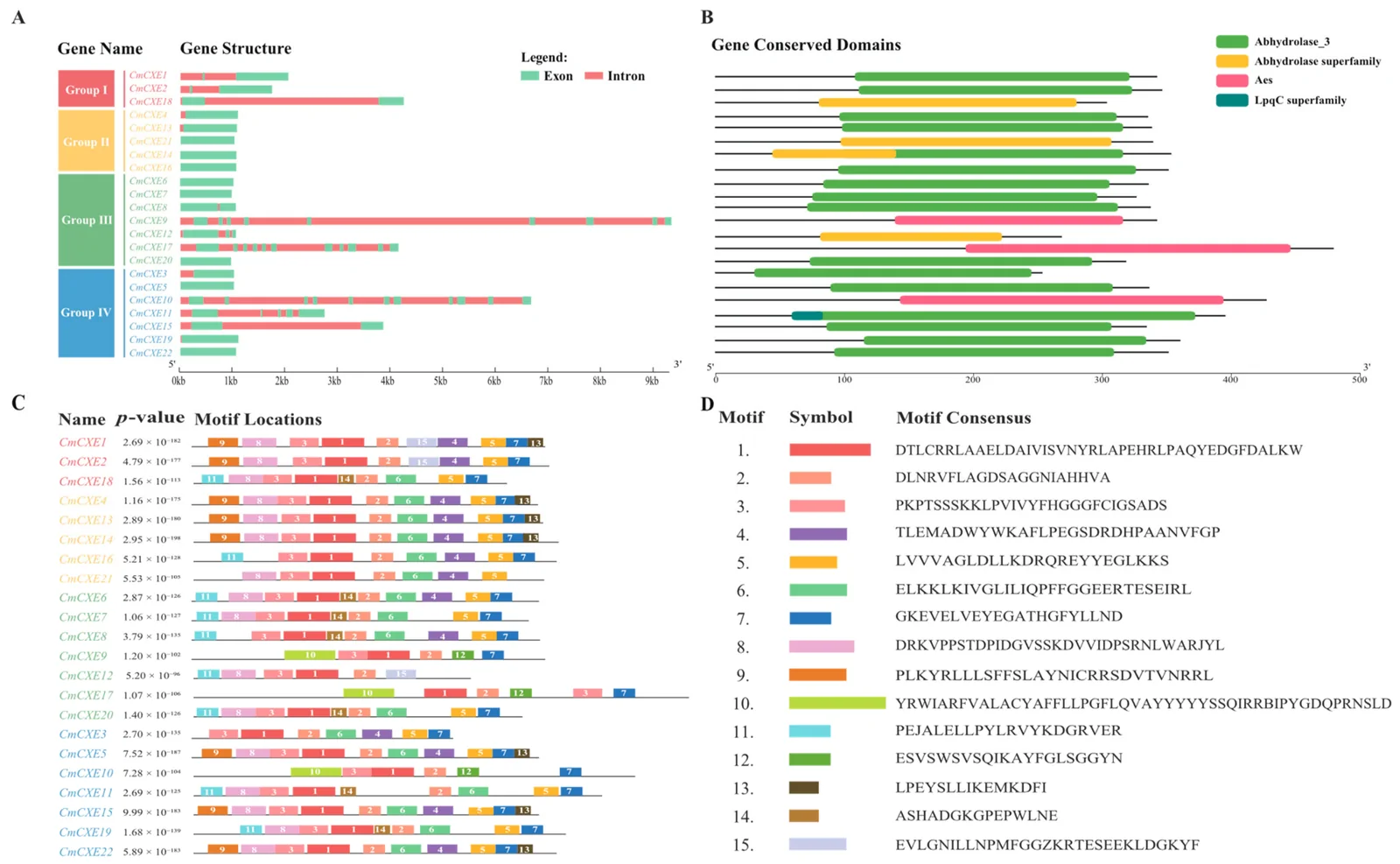
Gene structure, protein domain architecture, and conserved motif analysis of the melon CmCXE gene family. (**A**) Exon–intron structures of the 22 CmCXE genes. Green boxes represent exons; red boxes represent introns, with lengths displayed proportionally. Gene names and their phylogenetic group assignments (Groups I–IV) are indicated on the left. (**B**) Conserved domain architecture of CmCXE proteins. Abhydrolase_3 (green), Abhydrolase superfamily (yellow), Ase (dark pink), and LpqC superfamily (dark blue) domains are shown; black segments indicate non-domain regions. (**C**) Distribution of 15 conserved motifs across the 22 CmCXE protein sequences. (**D**) Amino acid sequences of the 15 conserved motifs, as predicted by MEME; motif numbers correspond to those in Panel (**C**).

To further characterize the CXE family, we analyzed gene structures and conserved motifs. Gene structure analysis revealed diverse exon–intron organizations among the 22 CmCXE genes: eight were intronless, five contained one intron, and nine harbored two or more introns. Intron distribution correlated with phylogenetic groupings, with multi-intron genes predominantly clustered in Groups III and IV, whereas Groups I and II contained few or no introns (Figure 3A).

Protein domain analysis confirmed that all CmCXE proteins contain α/β hydrolase superfamily domains, albeit with variable domain compositions (Figure 3B; detailed in Table S3). Conserved motif analysis identified 15 motifs (Motifs 1–15) (Figure 3C,D and Figure S4). Motifs 1–3 were present in all 22 members and constitute the catalytic core, containing the characteristic esterase active-site motif GXSXG (harboring the catalytic serine) and the oxyanion-hole motif HGGG. Motifs 4–8 were present in 13–20 members, whereas Motifs 9–15 showed group-specific distribution, predominantly in Groups III and IV. Overall, motif composition patterns were consistent with phylogenetic groupings, supporting the reliability of the phylogenetic classification.

### 3.4. Cis-Acting Element Analysis of the Melon CmCXE Gene Family

**Figure 4:**
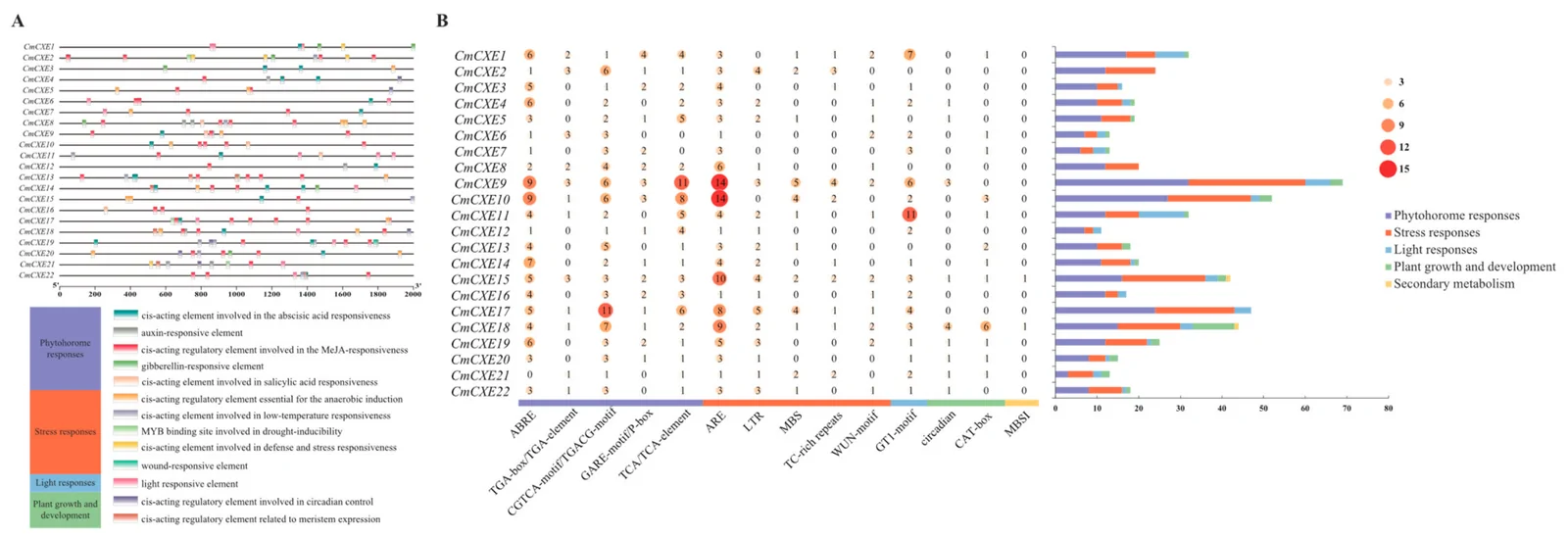
Cis-acting element analysis of the melon *CmCXE* gene family. (**A**) Distribution of cis-acting elements in the promoter regions (2000 bp upstream of the start codon, positive strand) of the 22 *CmCXE* genes. The exact positions of 13 core regulatory elements on the positive strand are shown; different colors and shapes represent different element types. (**B**) Bubble plot showing the copy-number distribution of cis-acting elements based on full-length sequences. Prediction was performed on the full-length nucleotide sequences of all 22 genes, and 14 representative elements were selected (MBSI added relative to Panel (**A**). Elements on both strands at identical positions were merged during statistical analysis. Bubble size is proportional to element copy number. The bar chart on the right shows the distribution of five element categories (phytohormone response, stress response, light response, plant growth and development, and secondary metabolism) in each gene.

Beyond coding sequences, we investigated the cis-acting regulatory elements in CmCXE gene promoters. To investigate the transcriptional regulatory mechanisms of the CmCXE genes, cis-acting elements in their promoter regions (2000 bp upstream of the start codon) were analyzed. Thirteen core cis-acting elements were identified and classified into four categories: hormone-responsive, stress-responsive, growth- and development-related, and light-responsive. Methyl jasmonate (MeJA)-responsive elements (CGTCA-motif) were the most abundant (57 copies across 21 genes), followed by abscisic acid (ABA)-responsive elements (ABRE; 36 copies across 18 genes) and anaerobic induction elements (ARE; 20 copies across 13 genes). Most members contained multiple CGTCA-motif copies, with *CmCXE2*, *CmCXE13*, and *CmCXE17* each harboring five copies (Figure 4A). The detailed positions and functional annotations of all identified cis-acting elements are provided in Table S4.

Complementary full-length sequence analysis identified one additional element, MBSI, which is involved in flavonoid biosynthesis and is located outside the 2000 bp proximal promoter window, bringing the total to 14 elements in five functional categories; distribution patterns were consistent with the promoter-based analysis (Figure 4B). Notably, members of Groups III and IV (e.g., *CmCXE9*, *CmCXE10*, *CmCXE17*) harbored both MeJA- and salicylic acid-responsive elements, suggesting their potential involvement in aroma metabolism through hormone signaling pathways.

### 3.5. Protein Structure Prediction of the Melon CmCXE Gene Family

**Figure 5:**
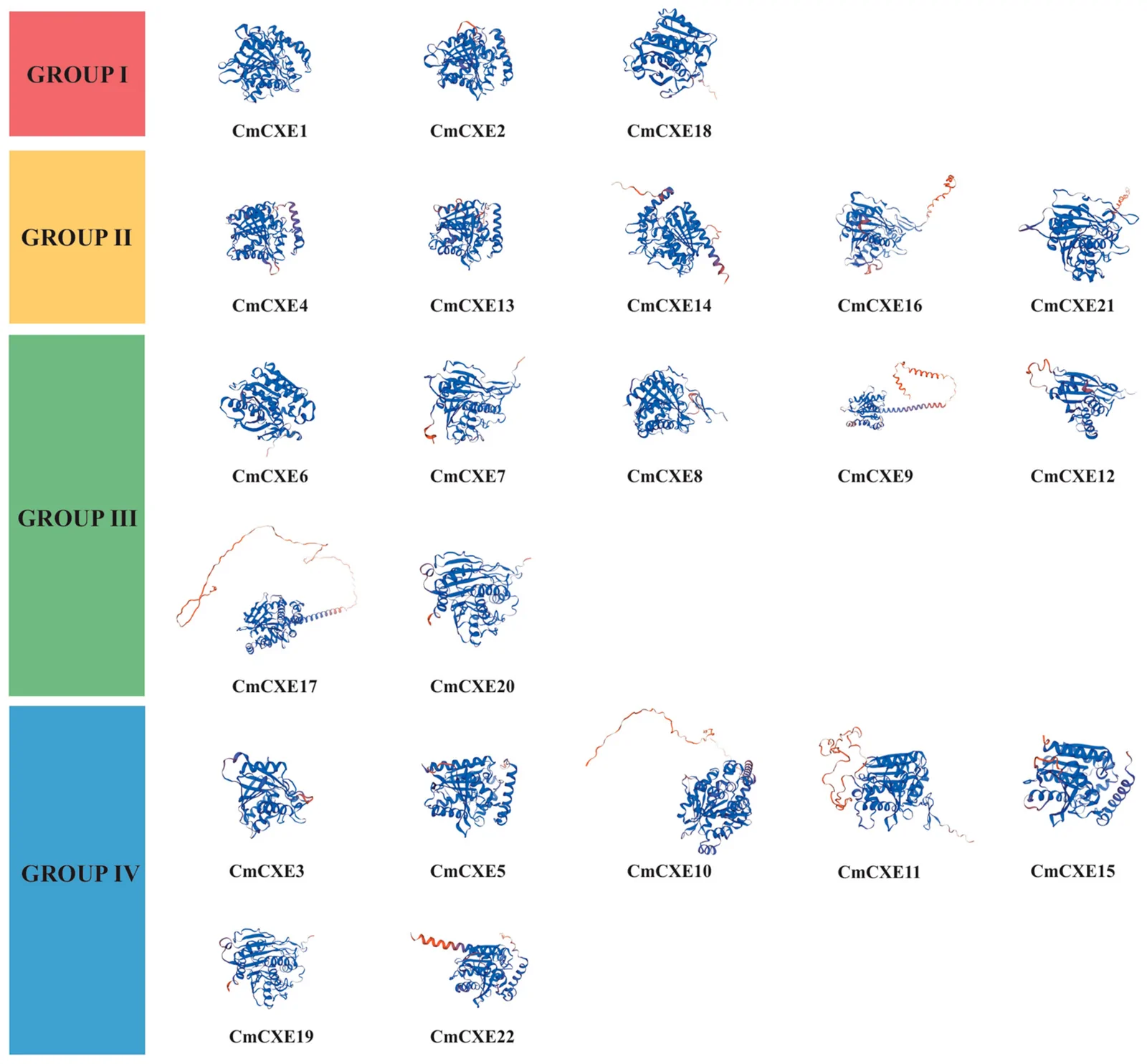
Predicted three-dimensional structures of melon CmCXE proteins. Three-dimensional structures of CmCXE proteins predicted using the AlphaFold2 module on the SWISS-MODEL server. Colors indicate prediction confidence (pLDDT): dark blue, high confidence (pLDDT > 90); light blue, moderate confidence (70 < pLDDT ≤ 90); yellow, low confidence (50 < pLDDT ≤ 70); red, very low confidence (pLDDT ≤ 50), indicating unreliable structural predictions in these regions.

The three-dimensional structures of the 22 CmCXE proteins were predicted using the AlphaFold2 module on the SWISS-MODEL server (Figure 5). Most CmCXE proteins exhibited the characteristic α/β hydrolase fold, consisting of a central β-sheet surrounded by α-helices, with secondary structural elements primarily comprising α-helices, β-sheets, and random coils. Comparative analysis within each phylogenetic group revealed that members of the same group generally shared conserved overall folds, although variations were observed in loop regions. For example, *CmCXE4* and *CmCXE13* (Group II), *CmCXE7* and *CmCXE20* (Group III), and *CmCXE5*, *CmCXE15*, and *CmCXE22* (Group IV) displayed highly similar spatial architectures. This structural conservation within groups suggests a conserved structural basis and potential functional similarity among closely related CmCXE members.

### 3.6. Predicted Functional Interaction Module of the Carboxylesterase Family

**Figure 6:**
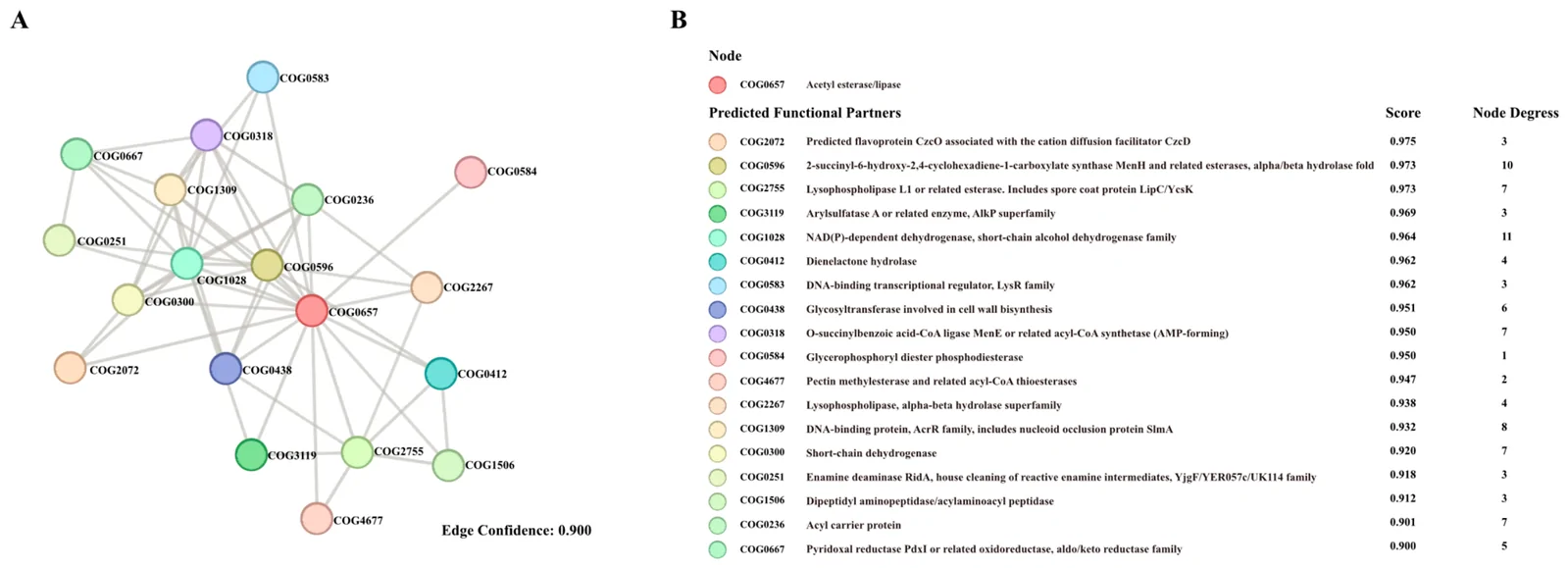
Predicted functional interaction module of the carboxylesterase family. (**A**) Interaction network of the carboxylesterase family (COG0657). Nodes represent predicted interacting proteins; node size indicates connectivity degree within the network, and lines represent interactions. Different colors represent distinct functional categories (esterases/lipases, oxidoreductases, other hydrolases, and transcriptional regulators). (**B**) Detailed list of interacting proteins. From left to right: COG code, functional description, and interaction score (higher scores indicate more reliable interactions). Color coding corresponds to node colors in Panel (**A**). The network was constructed using the STRING database (v12.0) with a high confidence threshold of 0.9.

To explore the potential functional partners of the CmCXE proteins, a PPI network was constructed using the STRING database (v12.0) with the carboxylesterase family (COG0657) as the query and a high confidence threshold of 0.9. A total of 18 predicted functional partners were identified, all with interaction scores ≥ 0.900 (Figure 6).

The interacting partners could be classified into four functional categories. (i) Esterases and lipases (COG0596, COG2755, COG2267, COG3119), including MenH-related esterases, lysophospholipases, and arylesterases, which share the α/β hydrolase fold with CXE proteins and may participate cooperatively in ester metabolism. (ii) Oxidoreductases (COG2072, COG1028, COG0300, COG0667), comprising short-chain dehydrogenases and flavoproteins, which may be involved in the redox reactions associated with volatile compound biosynthesis. (iii) Other hydrolases (COG0412, COG4677, COG0584, COG1506), including pectin methylesterase, glycerophosphoryl diester phosphodiesterase, and dipeptidyl aminopeptidase. (iv) Transcriptional regulators (COG0583, COG1309), including LysR-family and AcrR-family regulators, which may modulate the expression of ester-metabolism genes. The highest-confidence interactions were observed for COG2072 (predicted flavoprotein, score 0.975), COG0596 (MenH-related esterase, score 0.973), and COG2755 (lysophospholipase, score 0.973). Proteins with higher node degrees were predominantly esterases and oxidoreductases, suggesting their central positions in the network. Collectively, this conserved interaction module suggests that CmCXE proteins may function in concert with esterases, oxidoreductases, and transcriptional regulators to regulate ester metabolism and, potentially, aroma formation. Experimental validation in melon will be required to confirm these predicted interactions.

### 3.7. Expression Pattern Analysis of the Melon CmCXE Gene Family

**Figure 7:**
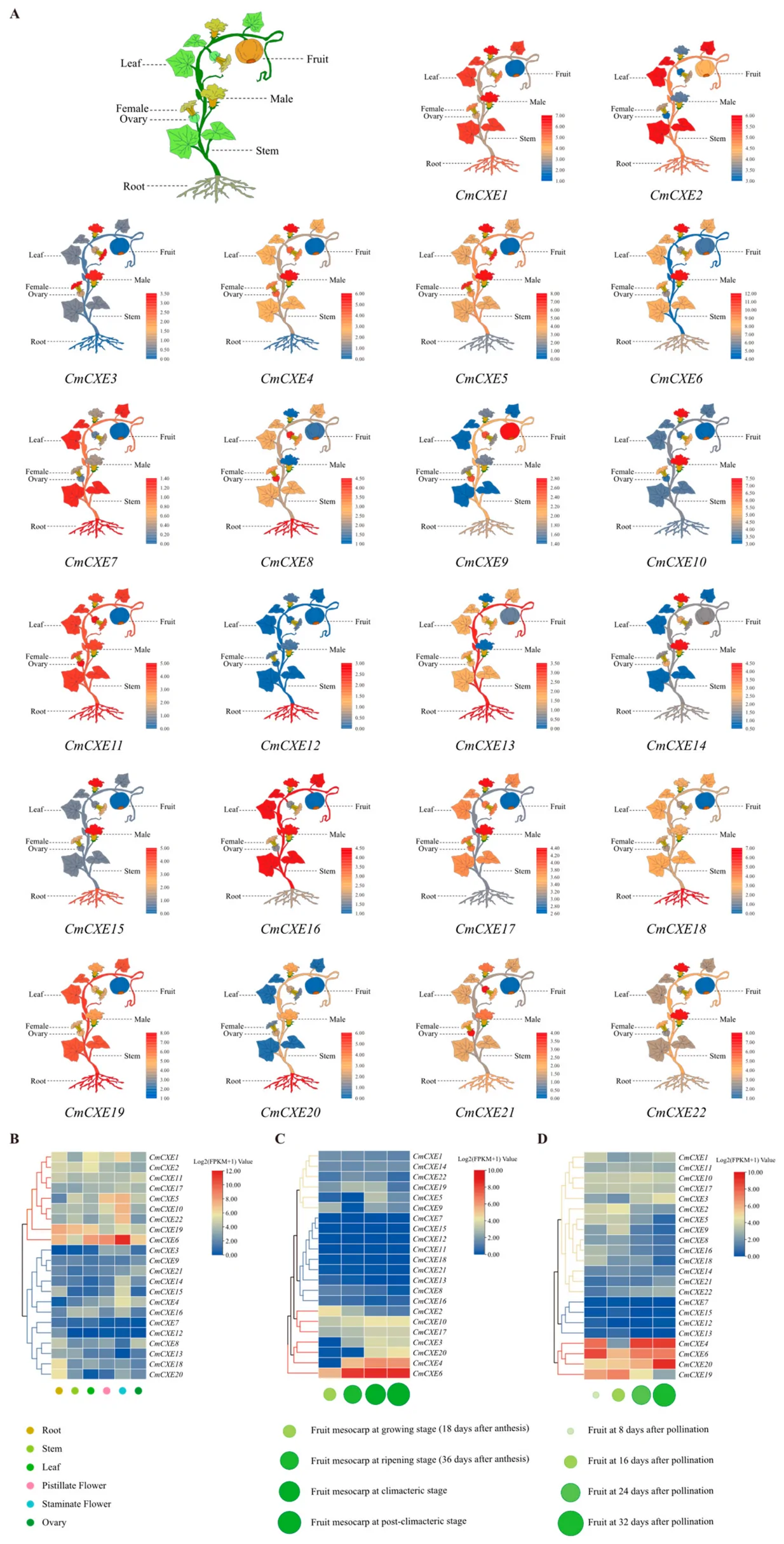
Expression pattern analysis of the melon *CmCXE* gene family. (**A**) Integrated heatmap of CmCXE gene expression across seven tissues/organs, combining tissue-specific data (PRJNA803327) with fruit mesocarp at the growing stage (18 days after anthesis, PRJNA543288). Expression values were row-normalized for each gene to show relative expression patterns. (**B**) Expression profiles of CmCXE genes in different melon tissues (PRJNA803327): root, stem, leaf, pistillate flower, staminate flower, and ovary. (**C**) Expression dynamics of CmCXE genes during fruit mesocarp development (PRJNA543288): 18 days after anthesis, 36 days after anthesis, climacteric stage, and post-climacteric stage. (**D**) Expression profiles of CmCXE genes at four fruit developmental time points after pollination (PRJNA300582): 8, 16, 24, and 32 days. All expression values were log_2_(FPKM + 1)-transformed. PRJNA803327 and PRJNA543288 were generated from cv. Hetao, and PRJNA300582 from cv. Gotgam (*Cucumis melo* var. *makuwa*). The unnormalized raw FPKM values for all datasets are available in Table S5.

To comprehensively investigate the expression patterns of the CmCXE genes, FPKM expression values were obtained from the CuGenDBv2 database based on the melon reference genome (DHL92 v4.0). Three transcriptome datasets were used: tissue-specific expression in root, stem, leaf, pistillate flower, staminate flower, and ovary (PRJNA803327); four developmental stages of fruit mesocarp (PRJNA543288); and four fruit developmental stages after pollination (PRJNA300582). All expression values were transformed by log_2_(FPKM + 1), and clustering heatmaps were constructed using TBtools (v1.098769; Figure 7B–D; Table S5).

To provide an integrated view of tissue-specific expression, a combined heatmap was generated by merging the six tissue types from PRJNA803327 with the fruit mesocarp at the growing stage (18 days after anthesis) from PRJNA543288, resulting in seven tissues/organs: root, stem, leaf, pistillate flower, staminate flower, ovary, and fruit. Both datasets were generated from the same melon cultivar (cv. Hetao) and aligned to the DHL92 v4.0 reference genome, ensuring comparability. Expression values were row-normalized for each gene (i.e., scaled relative to the maximum expression of that gene across all samples) to visualize relative expression patterns across tissues (Figure 7A). The results revealed diverse tissue preferences among the CmCXE genes: nine genes showed the highest relative expression in staminate flowers, seven in roots, two in ovaries, and one each in leaves, pistillate flowers, fruit, and stems. Notably, *CmCXE9* exhibited the highest relative expression in fruit, while *CmCXE3* showed flower-specific expression with the highest levels in both pistillate and staminate flowers. Eleven genes showed overall higher relative expression in reproductive organs (flowers, fruit, and ovary), whereas the remaining eleven genes were more highly expressed in vegetative organs (roots, stems, and leaves).

According to the clustering results of Figure 7B, the expression levels of CmCXE genes in different tissues could be clearly divided into two groups. Genes in the high-expression group showed strong expression signals in multiple tissues, among which *CmCXE6* and *CmCXE19* exhibited particularly high transcript abundance. In addition, *CmCXE1*, *CmCXE5*, *CmCXE10*, and *CmCXE22* also showed relatively high expression, with prominent expression levels detected in floral organs (detailed expression values are listed in Table S5). In contrast, *CmCXE7* and *CmCXE12*, belonging to the low-expression group, exhibited markedly lower transcript abundance in reproductive tissues and fruit developmental stages, with only weak expression detected in vegetative tissues (log_2_(FPKM + 1) = 0.26–1.83). Some other low-expression genes only showed faint expression in roots and staminate flowers, suggesting that these genes may have functional redundancy or specific spatiotemporal expression characteristics.

Figure 7C shows the expression patterns of CmCXE genes during fruit mesocarp development. Based on the clustering heatmap, these genes were divided into high-, moderate-, and low-expression groups. The high-expression group was mainly represented by *CmCXE4* and *CmCXE6*. Most members in this group showed high expression at the climacteric and post-climacteric stages, and their expression levels gradually increased with mesocarp development, whereas *CmCXE2* showed a declining expression pattern across mesocarp development (from 4.43 at the growing stage to 1.16 at the post-climacteric stage). Corresponding quantitative data are provided in Table S5. Members in the moderate-expression group maintained relatively stable expression throughout all stages. Genes in the low-expression group showed almost no expression at all mesocarp developmental stages, indicating that they may not participate in the regulation of mesocarp development.

The expression profiles of CmCXE genes at 8, 16, 24, and 32 days after pollination are shown in Figure 7D. Similarly, all genes were clustered into three groups according to their expression abundance. The high-expression group was composed of *CmCXE4*, *CmCXE6*, *CmCXE19*, and *CmCXE20*, with significantly higher expression levels than other genes: *CmCXE6* peaked at 8 days and then slightly declined; *CmCXE19* reached its maximum at 16 days, followed by a sharp drop; *CmCXE4* declined at 16 days after an initial peak at 8 days, then recovered and remained high at 24–32 days; while *CmCXE20* showed continuous upregulation and surged to the highest level at 32 days. The moderate-expression group included *CmCXE1*, *CmCXE2*, *CmCXE3*, *CmCXE5*, *CmCXE10*, *CmCXE11*, and *CmCXE17*, which maintained stable expression levels without dramatic fluctuations. The low-expression group was composed of *CmCXE7*, *CmCXE12*, *CmCXE13*, and *CmCXE15*, maintaining very low expression across almost all periods, and only faint expression signals could be detected in individual stages. Detailed expression values are shown in Table S5.

### 3.8. Subcellular Localization of the Melon CmCXE7 Protein

Based on our laboratory’s previous transcriptomic study identifying *CmCXE7* as a cold-responsive esterase gene in melon fruit (see Supplementary materials), this gene was selected for functional investigation. To investigate its in vivo function, transgenic tomato plants overexpressing *CmCXE7* under the control of the CaMV 35S promoter were generated. To determine the subcellular localization of *CmCXE7*, a *CmCXE7*-GFP fusion construct was transiently expressed in *Nicotiana benthamiana* leaves.

**Figure 8:**
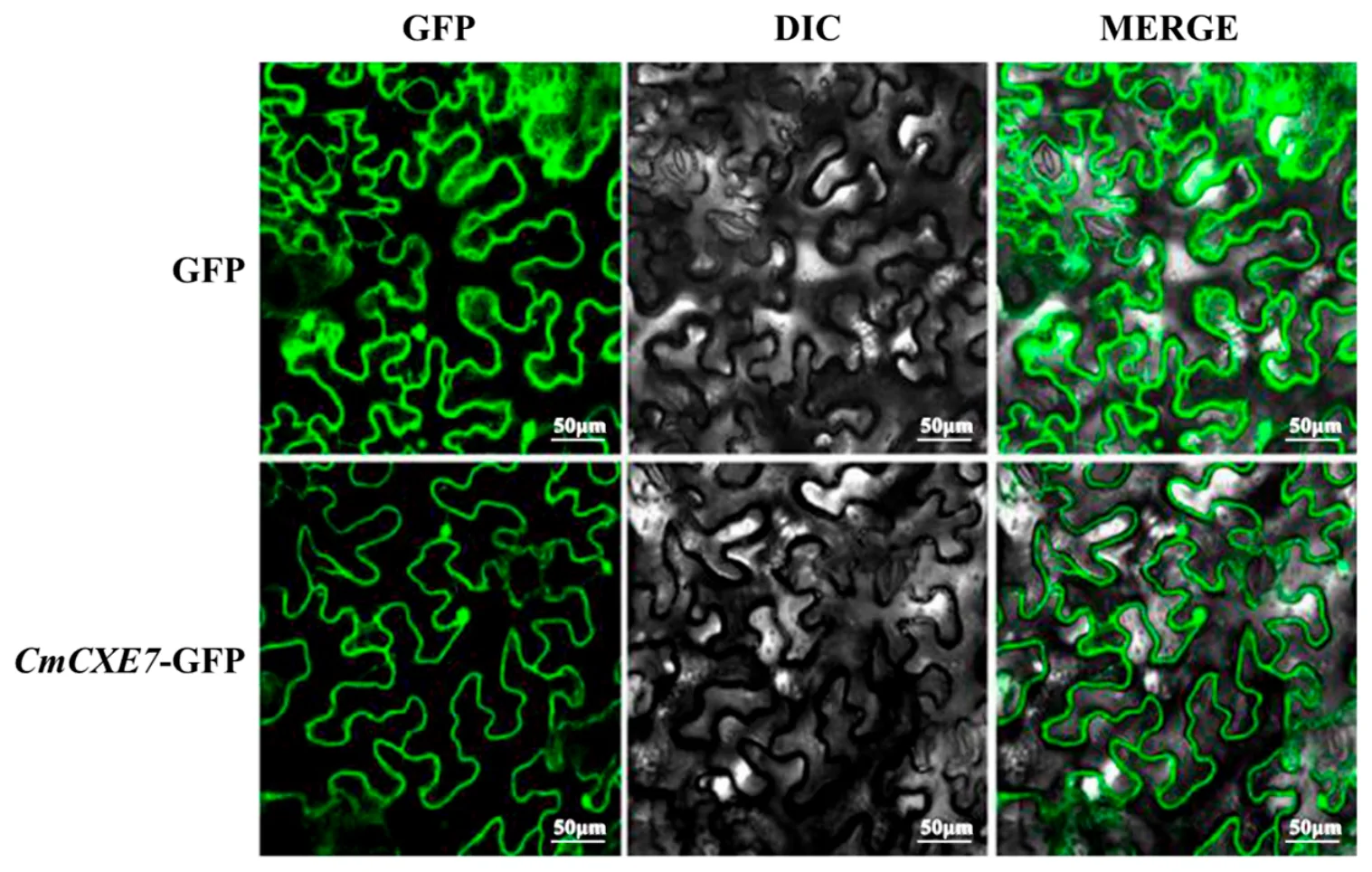
Subcellular localization of *CmCXE7* in *Nicotiana benthamiana* leaves. The *CmCXE7*-GFP fusion protein was transiently expressed in *N. benthamiana* epidermal cells and observed by laser scanning confocal microscopy. GFP fluorescence (green), bright-field (DIC), and merged images are shown. Scale bars = 50 μm.

WoLF PSORT prediction suggested that *CmCXE7* was primarily localized in the cytoplasm (score: 11), with lower scores for peroxisomes (score: 2) and endoplasmic reticulum/vacuole (score: 1). However, experimental observation showed that the *CmCXE7*-GFP fluorescence signal was predominantly concentrated at the plasma membrane, with a weak signal also detected in the nucleus (Figure 8). These results indicate that *CmCXE7* localizes to both the plasma membrane and the nucleus, which differs from the in silico prediction and suggests that *CmCXE7* may function at the cell periphery and in the nucleus.

### 3.9. Metabolomic Investigation of CmCXE7 Function in Transgenic Tomato

**Figure 9:**
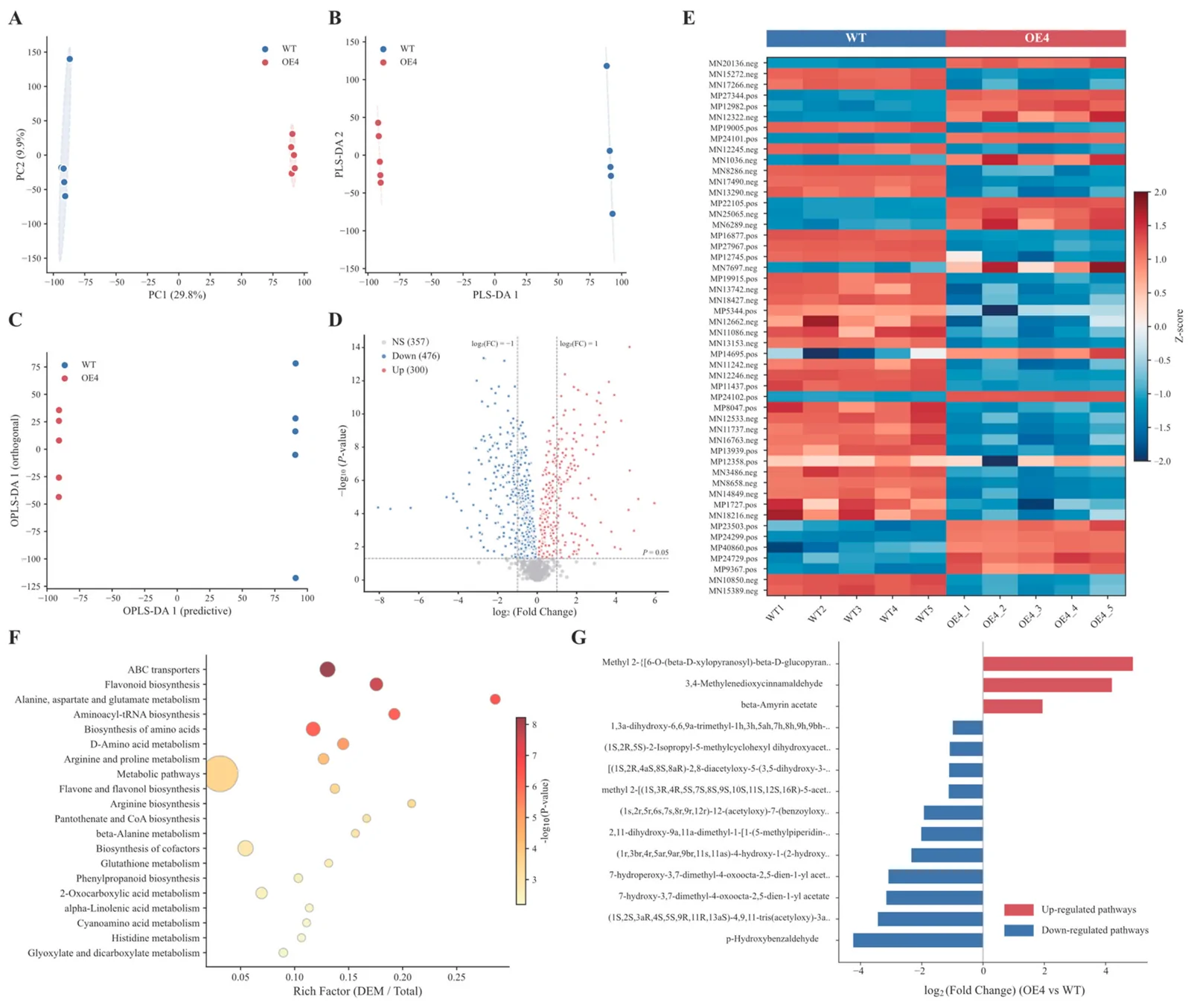
Non-targeted metabolomic analysis of OE4 and WT tomato fruits. (**A**) Principal component analysis (PCA) score plot showing the separation between OE4 and WT groups. (**B**) Partial least squares discriminant analysis (PLS-DA) score plot. (**C**) Orthogonal partial least squares discriminant analysis (OPLS-DA) score plot. (**D**) Volcano plot of DAMs between OE4 and WT; red and blue dots represent significantly up- and down-regulated metabolites (VIP > 1, *p* < 0.05), respectively, and grey dots represent non-significant metabolites. (**E**) Heatmap of ester- and aroma-related DAMs with |log_2_ (fold change)| > 1; the color scale represents the relative abundance of each metabolite in OE4 and WT, with red indicating higher abundance and blue indicating lower abundance. (**F**) KEGG pathway enrichment bubble plot of DAMs; the size of each bubble represents the number of DAMs enriched in the corresponding pathway, and the color represents the significance level (*p* value). (**G**) KEGG pathway enrichment bar chart showing the top enriched pathways. Metabolite names are partially truncated due to space constraints; complete names and details are provided in Table S8.

To experimentally investigate the potential role of *CmCXE7* in ester metabolism, non-targeted metabolomic profiling was performed on ripe fruits of the OE4 and WT controls (Supplementary Metabolomics Analysis Results). Quality control metrics, including TIC overlap, QC sample correlation, and RSD distribution, are summarized in Figure S6, confirming the reliability of the metabolomic data. A total of 894 annotated metabolites were detected across both ion modes (531 in positive mode, 363 in negative mode). Of these, 776 were DAMs between OE4 and WT based on VIP > 1 and *p* < 0.05, including 300 up-regulated and 476 down-regulated metabolites (Figure 9D; Table S6). Following Benjamini–Hochberg FDR correction, 655 of the 776 DAMs retained significance at FDR < 0.05, while the remaining 121 showed FDR values between 0.05 and 0.18 (Table S7). Principal component analysis (PCA) and orthogonal partial least squares discriminant analysis (OPLS-DA) showed clear separation between the OE4 and WT groups (Figure 9A–C). For the positive ion mode, the PLS-DA model showed R2X (cum) = 0.39, R2Y (cum) = 1.00, and Q2 (cum) = 0.933, and the OPLS-DA model showed R2X (cum) = 0.39, R2Y (cum) = 1.00, and Q2 (cum) = 0.904; permutation tests (200 permutations) yielded Q2 intercepts of 0.61 and 0.01, respectively, indicating robust models without overfitting (full statistics for both ion modes in Table S6).

Classification of the DAMs based on their superclass revealed that lipids and lipid-like molecules (187 metabolites), organic acids and derivatives (99), phenylpropanoids and polyketides (96), organoheterocyclic compounds (66), and organic oxygen compounds (60) were the most represented categories.

Given the established role of CXE proteins in ester hydrolysis, we specifically examined ester- and aroma-related metabolites. Among the 32 ester-related DAMs, 14 showed |log_2_ (fold change)| > 1, including 3 up-regulated and 11 down-regulated metabolites in OE4 (Figure 9E; Table S8) of these 14, 11 retained significance at FDR < 0.05; the remaining 18 ester-related DAMs with |log_2_ FC| ≤ 1 are included in the complete DAM list in Table S7. Notably, several volatile ester-related compounds were significantly reduced in OE4, such as 7-hydroxy-3,7-dimethyl-4-oxoocta-2,5-dien-1-yl acetate (log_2_ FC = −3.17) and (1S,2R,5S)-2-isopropyl-5-methylcyclohexyl dihydroxyacetate (log_2_ FC = −1.10), whereas two acetate conjugates were up-regulated. The predominance of down-regulated esters is consistent with the predicted function of *CmCXE7* as a putative carboxylesterase that may hydrolyze ester compounds. It should be noted that these metabolomic data were obtained by UHPLC-MS, which detects overall ester-related metabolites but does not specifically quantify volatile esters; GC-based approaches will be needed to confirm changes in the volatile fraction. From a postharvest perspective, the accumulation of volatile esters is a hallmark of advanced ripening; therefore, reduced ester accumulation in *CmCXE7*-overexpressing fruits may be associated with altered ripening-associated metabolism. While our data do not directly measure ripening time, the broad metabolic remodeling observed in OE4 fruits, including changes in phenylpropanoid and flavonoid pathways, suggests that *CmCXE7* overexpression may influence ripening-associated metabolic pathways beyond ester metabolism alone.

KEGG pathway enrichment analysis mapped the DAMs to multiple metabolic pathways, with the most significantly enriched pathways being ABC transporters (*p* = 6.01 × 10^−9^), flavonoid biosynthesis (*p* = 2.42 × 10^−8^), and alanine, aspartate and glutamate metabolism (*p* = 2.59 × 10^−7^) (Figure 9F,G; Table S9). Detailed sample information for the metabolomic analysis is provided in Table S10. Notably, phenylpropanoid biosynthesis was also significantly enriched (*p* = 3.10 × 10^−3^), which is particularly relevant as this pathway provides precursors for volatile ester biosynthesis. Taken together, these metabolomic data demonstrate that *CmCXE7* overexpression alters the ester metabolic profile and related pathways in tomato fruits, providing experimental support for the role of *CmCXE7* in ester and aroma metabolism.

## 4. Discussion

### 4.1. Phylogenetic Grouping Predicts Functional Potential: The Ester-Hydrolysis Clade

The CXE gene family exhibits lineage-specific expansion and contraction across land plants, with family sizes ranging from 16 in apple to 40 in ‘Nanguo’ pear [57,58]. The 22 CmCXE genes identified in melon and 26 *CsCXE* genes in cucumber place Cucurbitaceae at the lower-to-middle end of this spectrum, comparable to the 20 members in *Arabidopsis* and pineapple [17,59]. What is more informative than family size, however, is the phylogenetic distribution of functionally validated CXE genes. Cross-species comparisons have shown that ester-hydrolyzing CXE genes characterized to date, including peach *PpCXE1*, tomato *SlCXE1*, and strawberry *FvCXE2/3*, cluster within a single well-supported clade [9,14,15]. In the phylogenetic classification of the present study, this clade corresponds to Group III.

In melon, Group III contains *CmCXE6*, *CmCXE7*, *CmCXE8*, *CmCXE9*, *CmCXE12*, *CmCXE17*, and *CmCXE20*. Among these, *CmCXE6*, *CmCXE17*, and *CmCXE20* show elevated expression during fruit ripening, with peak expression at the climacteric or post-climacteric stages (Figure 7), whereas *CmCXE7*, *CmCXE8*, and *CmCXE12* exhibit low or undetectable transcript levels in fruit. The concordance between phylogenetic placement in the ester-hydrolysis clade and ripening-associated expression patterns suggests that these ripening-induced Group III members may contribute to ester metabolism in melon fruit, analogous to their orthologs in peach [24], tomato [14], and strawberry [16], although direct functional validation remains required. The 16 orthologous syntenic pairs between melon and cucumber (Figure 2B) indicate that this Group III clade was already established in the common ancestor of Cucurbitaceae and has been evolutionarily conserved. Consistent with the deep divergence between Rosaceae and Cucurbitaceae, no syntenic relationship was detected between melon Group III genes and the functionally validated strawberry *FvCXE* genes (Figure S2), reinforcing that phylogenetic placement, rather than cross-family synteny, provides the more reliable framework for functional inference at this evolutionary distance.

### 4.2. A Conserved Catalytic Core Underpins Divergent Subcellular and Regulatory Strategies

All 22 CmCXE proteins retain the canonical GXSXG catalytic nucleophile motif within Motifs 1–3, and AlphaFold2 predictions confirm the characteristic α/β hydrolase fold—a central parallel β-sheet surrounded by α-helices (Figure 5). This structural conservation is remarkable given the substantial variation in protein length (253–479 aa) and auxiliary domain composition (Table 1; Figure 3B). The evolutionary implication is clear: the catalytic machinery for ester hydrolysis was fixed early and is under strong evolutionary constraint, while the N- and C-terminal extensions and auxiliary domains have evolved to mediate subcellular targeting, protein–protein interactions, and substrate specificity [11,59,60].

This “conserved core, variable periphery” architecture helps explain a striking finding of this study: the discrepancy between predicted and experimentally determined subcellular localization of *CmCXE7*. WoLF PSORT predicted cytoplasmic localization, but transient expression in *Nicotiana benthamiana* revealed a predominant plasma-membrane signal with a weak nuclear signal (Figure 8), although this interpretation is based on DIC morphology rather than marker co-localization. Classical ester-hydrolyzing CXE proteins such as peach *PpCXE1* are cytoplasmic, where they co-localize with ester biosynthetic enzymes [15]. Nuclear-localized carboxylesterases, however, have been reported in other plant systems—most notably cotton *GBCXE49*, which responds to alkaline stress and was experimentally confirmed to localize to the nucleus [11]. The nuclear localization of *CmCXE7*, combined with its specific induction under cold storage (our laboratory’s prior transcriptomic data), raises the possibility that this protein may participate in stress signal transduction in addition to ester catalysis. This hypothesis is consistent with the enrichment of MeJA- and salicylic acid-responsive elements in the promoters of Group III/IV genes (Figure 4).

### 4.3. Temporal Coupling of CmCXE Expression with Ester Accumulation Suggests Coordinated Regulation

Fruit aroma is determined by the dynamic balance between ester biosynthesis, catalyzed primarily by alcohol acyltransferases (AATs), and ester hydrolysis, catalyzed by CXEs [4,61]. In melon, the climacteric ripening period (24–32 days after pollination) coincides with the rapid accumulation of characteristic acetate and hexanoate esters [25]. The expression profiles of *CmCXE6*, *CmCXE17*, *and CmCXE20*, Group III members with ripening-associated expression, rise progressively during this window and peak at the post-climacteric stage (Figure 7C,D). This pattern is not what one would expect if CXEs acted purely as negative regulators of aroma: if their sole function were to degrade esters, high expression during the peak accumulation period would be counterproductive.

A more parsimonious interpretation, supported by studies in peach and strawberry, is that CXEs function in metabolic channeling and precursor recycling rather than simple catabolism [9,15,24]. By hydrolyzing a subset of esters, CXEs release alcohol moieties that can be re-esterified with different acyl-CoA donors, thereby reshaping the ester profile rather than simply reducing total ester content. This model is consistent with the observation in pineapple that *AcCXE4* and *AcCXE7* show negative correlations with total aroma accumulation but are nonetheless expressed during ripening [17]. In melon, the co-expression of Group III CmCXE genes with the climacteric ester burst may reflect a homeostatic mechanism that fine-tunes the ratio of acetate esters to other ester classes. Direct testing of this hypothesis will require paired transcriptomic and volatile metabolomic time-course data across melon ripening. Furthermore, the presence of MeJA-, abscisic acid-, and light-responsive cis-elements in the promoters of ripening-associated CmCXE genes (Table S4) suggests that their coordinated expression may be governed by upstream transcription factor networks; systematic mapping of these regulatory interactions, informed by recent advances in transcription factor atlas resources [62], will be an important direction for future work.

### 4.4. Metabolomic Investigation of CmCXE7: Evidence for Broad Metabolic Remodeling Beyond Ester Hydrolysis

It is worth noting that *CmCXE7* was not the most highly expressed Group III member during fruit ripening; *CmCXE6*, *CmCXE17*, and *CmCXE20* showed stronger ripening-associated expression (Section 3.7). *CmCXE7* was nevertheless prioritized for functional validation because it was independently identified as a cold-responsive esterase in our prior transcriptomic work, providing a direct functional clue that the higher-expressed candidates lacked. The highly expressed Group III members remain important targets for future investigation. The heterologous overexpression of *CmCXE7* in tomato provides direct in vivo evidence for its catalytic activity. The 776 DAMs (300 upregulated, 476 downregulated) between OE4 and WT (Figure 9) indicate that *CmCXE7* expression induces broad metabolic remodeling, not merely changes in a few ester species. Among the 32 ester-related DAMs, 14 showed |log_2_ (fold change)| > 1, including 3 upregulated and 11 downregulated metabolites, a directionality consistent with ester hydrolytic activity. This pattern mirrors the transient overexpression of strawberry *FvCXE2* and *FvCXE3* in strawberry fruits, which reduced the contents of (Z)-3-hexenyl acetate, methyl hexanoate, and methyl butyrate [9], and the stable overexpression of peach *PpCXE1*, which accelerated ester catabolism [15].

However, two aspects of the metabolomic data warrant careful interpretation. First, the majority of DAMs are not ester-related, with significant enrichment in ABC transporters, flavonoid biosynthesis, and phenylpropanoid biosynthesis (Figure 9F,G). This broad metabolic impact is unlikely to result from direct ester hydrolysis alone; it more plausibly reflects secondary consequences of altered hormone signaling or redox homeostasis, consistent with the nuclear localization of *CmCXE7* and its predicted interactions with oxidoreductases (Section 3.6). Second, the heterologous tomato system may not fully recapitulate the substrate specificity of *CmCXE7* in its native melon context—tomato and melon have distinct ester profiles, and the absence of melon-specific interacting proteins could alter enzymatic behavior. These caveats notwithstanding, the metabolomic data provide in vivo evidence consistent with a putative ester-hydrolyzing activity of *CmCXE7*. If further validated, manipulating *CmCXE7* activity may provide a strategy for modulating ester accumulation and thereby influencing ripening-associated aroma development.

### 4.5. Predicted Interaction Networks Reveal a Multi-Enzyme Module for Ester Homeostasis

The STRING analysis, using the conserved carboxylesterase family (COG0657) as the query, predicted a functional interaction module of esterases, lipases, and oxidoreductases (Figure 6). Although this network is based on orthologous interactions across species rather than melon-specific experimental data, it provides a useful framework for hypothesis generation, including members of the COG0596 (abhydrolase), COG2755 (lysophospholipase), and COG2267 (MenH-related esterase) families. This predicted interactome is consistent with emerging evidence that ester metabolism in plants is organized into multi-enzyme metabolons rather than carried out by isolated enzymes [63]. In pumpkin, CXE proteins have been implicated in stress-associated metabolic pathways involving oxidoreductases [64], and in tomato, the esterase *SlCXE1* is part of a larger flavor volatile network that includes alcohol dehydrogenases and hydroperoxide lyases [14].

The enrichment of oxidoreductases in the CmCXE interactome is particularly noteworthy in light of the cold-stress phenotype of *CmCXE7*. Cold stress induces reactive oxygen species (ROS) accumulation and lipid peroxidation, generating a variety of ester and aldehyde products [65]. CXE-mediated hydrolysis of lipid-derived esters could feed substrates into the oxylipin pathway, linking aroma metabolism to stress signaling. This connection provides a mechanistic hypothesis for why *CmCXE7* is specifically induced by cold storage and why its overexpression in tomato alters flavonoid and phenylpropanoid pathways, both of which are involved in antioxidant defense. Experimental validation of these interactions via yeast two-hybrid or co-immunoprecipitation [66,67] will be essential to test this model.

### 4.6. Limitations and Future Directions

Several limitations of this study should be acknowledged. First, the functional validation of *CmCXE7* was performed in a heterologous tomato (*Solanum lycopersicum*) system and cannot confirm substrate specificity or regulatory function in melon (*Cucumis melo*); tomato and melon also differ in their characteristic ester profiles [10,68]. Although two positive transgenic lines (OE2 and OE4) were obtained, only OE4 was used for metabolomics due to its higher transgene expression, while OE2 was excluded because of limited seed availability and unclear phenotypic segregation in the T1 generation; no empty-vector control or transgene quantitative real-time PCR (qRT-PCR) confirmation was performed, so tissue-culture and insertion-site effects cannot be excluded. Subcellular localization was additionally determined in *Nicotiana benthamiana* leaves without organelle markers, so the weak nuclear signal remains tentative and may differ in native melon tissue. Second, all expression data were derived from public CuGenDBv2 transcriptome datasets rather than experimental measurements, representing two melon cultivars (Hetao and Gotgam) plus a third cultivar (‘Huaitian No. 1’) in our prior work; the integrated tissue heatmap combined two independent projects and may contain batch effects, and row normalization for visualization obscures absolute expression differences (raw FPKM values in Table S5), so key candidates require future qRT-PCR validation. Third, the metabolomic data were obtained by UHPLC-MS, which detects overall ester-related metabolites but does not specifically quantify volatile esters, so GC-MS or SPME-GC-MS would be needed to confirm volatile-fraction changes; all annotations are MS/MS-based (MSI level 2) without authentic-standard confirmation. Fourth, no direct ripening phenotypic measurements (firmness, color, ethylene production, weight loss) were performed, so the relevance to ripening and postharvest storage is inferred from metabolism alone; the gene family identification used Ensembl Plants paralogue prediction from a single query rather than a standard HMMER + BLASTP pipeline, which may miss highly divergent members despite domain verification; and the proposed role of *CmCXE7* in stress signaling remains speculative, based only on indirect evidence.

Future work should prioritize: (i) in vitro enzyme assays to determine the substrate preference and kinetic parameters of *CmCXE7* against melon-characteristic esters [69]; (ii) melon fruit transient overexpression or virus-induced gene silencing (VIGS) to validate *CmCXE7* function directly in the native melon context [70]; (iii) functional characterization of the high-expression Group III candidates (*CmCXE6*, *CmCXE17*, *CmCXE20*); (iv) qRT-PCR validation of key candidate genes across melon cultivars and developmental stages; and (v) protein interaction studies to confirm the predicted ester-homeostasis module. We acknowledge that the current tomato-based evidence remains preliminary until confirmed in melon.

## 5. Conclusions

This study provides a genome-wide identification of the CXE gene family in melon, identifying 22 members across four phylogenetic groups. The integration of phylogenetic, structural, expression, and metabolomic evidence converges on two key conclusions. First, Group III genes (*CmCXE6*, *CmCXE7*, *CmCXE8*, *CmCXE9*, *CmCXE12*, *CmCXE17*, *CmCXE20*) emerge as candidate regulators of ester metabolism during fruit ripening, based on their phylogenetic placement in the functionally validated ester-hydrolysis clade, their ripening-associated expression patterns, and their structural conservation. Second, *CmCXE7* represents a distinct functional category: a plasma membrane-localized (with weak nuclear signal), cold-responsive CXE whose heterologous overexpression induces broad metabolic remodeling, including changes consistent with ester hydrolysis, suggesting a role at the interface of aroma metabolism and stress signaling, pending further functional validation in melon. These findings fill a gap in Cucurbitaceae CXE research and provide candidate genes for molecular breeding aimed at preserving melon aroma quality during postharvest cold storage.

## Figures and Tables

**Table 1:** Identification, physicochemical properties, and subcellular localization prediction of the melon *CmCXE* gene family.

CmCXEs	Gene Locus ID	Length (bp)	ORF (aa)	MW (kDa)	PI	Aliphatic Index	Instability	II	GRAVY	Predicted Locations	TD	SP
*CmCXE1*	MELO3C015880	2153	342	38.69	6.27	89.18	unstable	40.7	−0.211	cyto:13, E.R._vacu:1	0	No
*CmCXE2*	MELO3C015362	2180	346	38.80	5.85	89.57	stable	39.44	−0.191	chlo:9, cyto:3, plas:1, vacu:1	0	No
*CmCXE3*	MELO3C027137	956	253	28.58	5.51	92.81	unstable	48.36	−0.105	cyto:7, cysk:5, extr:1, golg:1	0	No
*CmCXE4*	MELO3C008100	1102	335	37.62	6.46	81.19	unstable	48.79	−0.218	chlo:6, cyto:5, nucl:1, plas:1, cysk:1	0	No
*CmCXE5*	MELO3C026557	1774	336	38.03	6.1	86.19	unstable	41.35	−0.275	nucl:5, cyto:4, chlo:3, mito:1, pero:1	0	No
*CmCXE6*	MELO3C011389	1283	336	37.15	5.52	94.61	unstable	44.41	−0.066	cyto:10, cysk:2, plas:1, E.R._vacu:1	0	No
*CmCXE7*	MELO3C010887	1156	326	35.97	5.14	90.61	stable	32.91	−0.099	cyto:11, pero:2, E.R._vacu:1	0	No
*CmCXE8*	MELO3C014690	1227	337	37.40	5.14	95.19	stable	37.4	−0.089	cysk:11, cyto:2, E.R._vacu:1	0	No
*CmCXE9*	MELO3C020397	9308	342	38.80	9.42	92.95	unstable	47.69	−0.099	cyto:8, cysk:3, nucl:2, E.R._vacu:1	2	No
*CmCXE10*	MELO3C023775	6961	427	47.62	6.72	95.9	unstable	45.54	−0.014	chlo:5, nucl:2, cyto:2, golg:2, mito:1, plas:1, vacu:1	1	No
*CmCXE11*	MELO3C016739	2916	395	44.11	5.32	95.8	stable	36.78	−0.342	cyto:10, cysk:2, chlo:1, E.R._vacu:1	0	No
*CmCXE12*	MELO3C032932	1048	268	29.82	7.12	93.47	unstable	52.84	−0.126	cyto:6, nucl:3, cysk:2, chlo:1, golg:1, E.R._vacu:1	0	No
*CmCXE13*	MELO3C022132	1080	338	38.14	6.26	86.6	stable	32.49	−0.121	chlo:12, mito:1.5, cyto_mito:1.5	0	No
*CmCXE14*	MELO3C022130	1126	353	39.93	6.52	83.46	unstable	48.82	−0.218	chlo:11, nucl:1, mito:1, plas:1	0	No
*CmCXE15*	MELO3C022125	4047	334	37.87	8.44	80.51	unstable	42.76	−0.202	chlo:12, mito:2	0	No
*CmCXE16*	MELO3C012228	1147	351	39.23	7.06	92.99	stable	35.23	−0.062	cyto:7, nucl:3, mito:2, plas:1, cysk:1	0	No
*CmCXE17*	MELO3C012201	4598	479	53.23	6.84	86.74	unstable	57.29	−0.108	chlo:6, nucl:3, mito:1.5, mito_plas:1.5, vacu:1, pero:1, golg:1	1	No
*CmCXE18*	MELO3C021904	4421	303	33.67	5.39	84.06	unstable	46.62	−0.225	cyto:7, chlo:6, plas:1	0	No
*CmCXE19*	MELO3C021903	1105	360	40.34	6.1	93.22	unstable	42.07	−0.082	mito:9, E.R.:2, chlo:1, vacu:1, golg:1	1	No
*CmCXE20*	MELO3C021901	1173	318	35.30	4.99	87.99	stable	32.38	−0.269	cyto:10, cysk:3, plas:1	0	No
*CmCXE21*	MELO3C022513	1019	339	37.39	6.83	90.94	unstable	43.89	−0.01	mito:6, chlo:5, plas:2, cyto:1	0	No
*CmCXE22*	MELO3C002695	1114	351	39.96	6.47	83.59	unstable	48.44	−0.31	nucl:8, cyto:5, mito:1	0	No

Abbreviations: ORF, open reading frame; aa, amino acid; MW, molecular weight; PI, isoelectric point; AI, Aliphatic index; II, Instability index; GRAVY, Grand average of hydropathicity; cyto, cytoplasm; nucl, nucleus; mito, mitochondrion; chlo, chloroplast; plas, plasma membrane; vacu, vacuole; golg, Golgi apparatus; pero, peroxisome; E.R., endoplasmic reticulum; extr, extracellular; cysk, cytoskeleton; E.R._vacu, endoplasmic reticulum or vacuole; cyto_mito, cytoplasm or mitochondrion; mito_plas, mitochondrion or plasma membrane; TD, Transmembrane domain; SP, Signal peptide. Values indicate the prediction reliability score, with higher scores representing higher probability of localization at the corresponding site. Multiple localizations suggest possible dual targeting or condition-dependent localization.

## Data Availability

The melon (*Cucumis melo* L., cv. DHL92, v4.0) and cucumber (*Cucumis sativus* L., cv. ChineseLong, v2.0) reference genomes and annotation files were obtained from the Cucurbit Genomics Database v2 (CuGenDBv2, http://cucurbitgenomics.org/v2/). The strawberry (*Fragaria vesca*) genome was downloaded from the Genome Database for Rosaceae (GDR, https://www.rosaceae.org/). Public transcriptome datasets used in this study are available in the NCBI Sequence Read Archive (SRA) under accession numbers PRJNA803327 (tissue-specific expression, cv. Hetao), PRJNA543288 (fruit mesocarp development, cv. Hetao), and PRJNA300582 (fruit development after pollination, cv. Gotgam). The complete metabolomic analysis dataset, including QC results, multivariate statistical outputs, peak area matrices, and differential metabolite lists, is provided as a Supplementary Compressed File (Supplementary Metabolomics Analysis Results), available for download from the article’s Supplementary Materials section.

## Supplementary Materials

The following supporting information can be downloaded at: https://www.mdpi.com/article/10.3390/plants15203144/s1, Supplementary tables: Table S1: Detailed information on 22 CmCXE genes in melon and 26 CsCXE genes in cucumber, including their original gene IDs. Table S2: Information on 35 FvCXE genes in strawberry, including their original gene IDs. Table S3: Statistical parameters of the 15 conserved motifs identified in this study. Table S4. Distribution of cis-acting elements in the 2000 bp promoter regions (upstream of the start codon) of the 22 CmCXE genes. Table S5: Expression levels of CmCXE genes in different tissues and fruit developmental stages of melon, including both log_2_(FPKM + 1) (normalized for heatmap visualization) and raw FPKM values. Table S6: Summary of differential metabolites and model validation statistics between WT and OE4 tomato fruits. Table S7: Complete list of 776 DAMs between OE4 and WT tomato fruits (VIP > 1, *p* < 0.05). Table S8: Ester/aroma-related DAMs between OE4 and WT tomato fruits (|log2FC| > 1). Table S9: KEGG pathway enrichment analysis of the 776 DAMs. Table S10: Sample information for the metabolomics analysis. Supplementary materials: Detailed experimental protocols and supplementary figures for the preliminary functional characterization of *CmCXE7*, including reverse transcription systems, PCR amplification systems and programs, vector digestion and homologous recombination systems, primer sequences, media formulations, lists of reagents and instruments used (Tables S11–S21), as well as unrooted phylogenetic tree (Figure S1), collinearity analysis (Figure S2), sequence logos of the 15 conserved motifs (Figure S3), multiple sequence alignment (Figure S4), preliminary functional validation (Figure S5), and quality control of the metabolomic profiling (Figure S6). Supplementary Metabolomics Analysis Results: Complete metabolomics analysis dataset for WT vs. OE4 tomato fruit comparison, including raw data processing, QC metrics, multivariate statistics, and pathway enrichment results.

## Abbreviations

The following abbreviations are used in this manuscript:

Abbreviation	Full Term
CXE	Carboxylesterase
VOC	Volatile organic compound
AAT	Alcohol acyltransferase
ML	Maximum likelihood
CuGenDB	Cucurbit Genomics Database
GDR	Genome Database for Rosaceae
CDD	Conserved Domain Database
GSDS	Gene Structure Display Server
pI	Isoelectric point
GRAVY	Grand average of hydropathicity
FPKM	Fragments per kilobase of transcript per million mapped reads
SRA	Sequence Read Archive
PPI	Protein–protein interaction
GFP	Green fluorescent protein
DIC	Differential interference contrast
UHPLC	Ultra-high-performance liquid chromatography
ESI	Electrospray ionization
PCA	Principal component analysis
PLS-DA	Partial least squares discriminant analysis
OPLS-DA	Orthogonal partial least squares discriminant analysis
DAMs	Differentially accumulated metabolites
VIP	Variable importance in projection
KEGG	Kyoto Encyclopedia of Genes and Genomes
HMDB	Human Metabolome Database
MeJA	Methyl jasmonate
ABA	Abscisic acid
ABRE	ABA-responsive element
ARE	Anaerobic induction element
WT	Wild type
OE	Overexpression
ROS	Reactive oxygen species
FDR	False discovery rate
JA	jasmonic acid
VIGS	virus-induced gene silencing
qRT-PCR	Quantitative real-time polymerase chain reaction
