# Supplementary material for: Genome-Wide Identification and Functional Characterization of the Carboxylesterase (CXE) Gene Family in Melon (Cucumis melo L.): CmCXE7 as a Cold-Responsive Regulator of Ester Metabolism
Source: Plants (Basel). 2026 Oct 14;15(20):3144. doi: 10.3390/plants15203144 (PMC13709046; doi:10.3390/plants15203144)
Supplement: Supplementary file 1 [file plants-15-03144-s001.zip › Supplementary Materials/Supplementary materials.pdf]

Supplementary Materials

Genome-Wide Identification and Functional Characterization of the Carboxylesterase (CXE) Gene Family in Melon (*Cucumis melo* L.): *CmCXE7* as a Cold-Responsive Regulator of Ester Metabolism

This supplementary material document consists of two parts:

Part I: Supplementary Figures (Figures S1–S4, S6)

This part contains the supplementary figures referenced in the main text, including the unrooted phylogenetic tree of CXE proteins from melon and cucumber (**Figure S1**), collinearity analysis between melon and strawberry CXE genes (**Figure S2**), sequence logos of the 15 conserved motifs (**Figure S3**), multiple sequence alignment of the 22 *CmCXE* proteins generated by GeneDoc (**Figure S4**), and quality control of the metabolomic profiling(**Figure S6**).

Part II: Supplementary Methods (Figure S5 and Tables S11–S21)

This part provides detailed experimental protocols for the preliminary functional characterization of *CmCXE7*, including the rationale for gene selection, gene cloning and vector construction, subcellular localization assays, and generation of transgenic tomato lines. The supplementary figure (**Figure S5**) documents the key experimental steps, and **Tables S11–S21** provide the detailed reaction systems, primer sequences, media formulations, and lists of reagents and instruments used in these experiments.

Part I: Supplementary Figures (Figures S1–S4, S6)

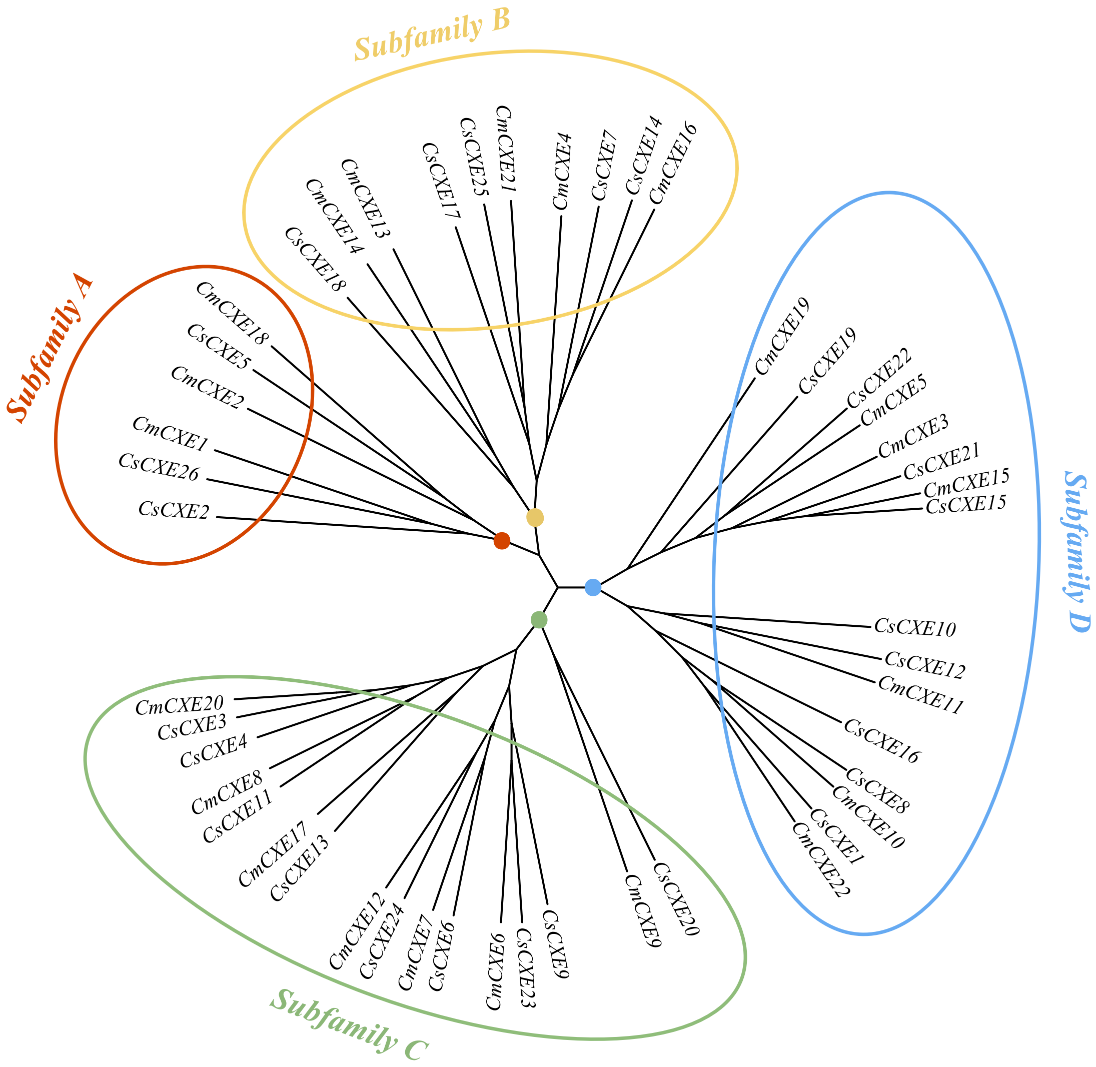

Figure S1. Unrooted phylogenetic tree of melon and cucumber CXE proteins.

Note: The tree was constructed using the Maximum Likelihood method with 1,000 bootstrap replicates. Bootstrap values (≥50%) are shown at nodes. The four clades are labeled Subfamilies A–D in the figure, which correspond to Groups I–IV as described in the main text (Subfamily A = Group I, Subfamily B = Group II, Subfamily C = Group III, Subfamily D = Group IV).

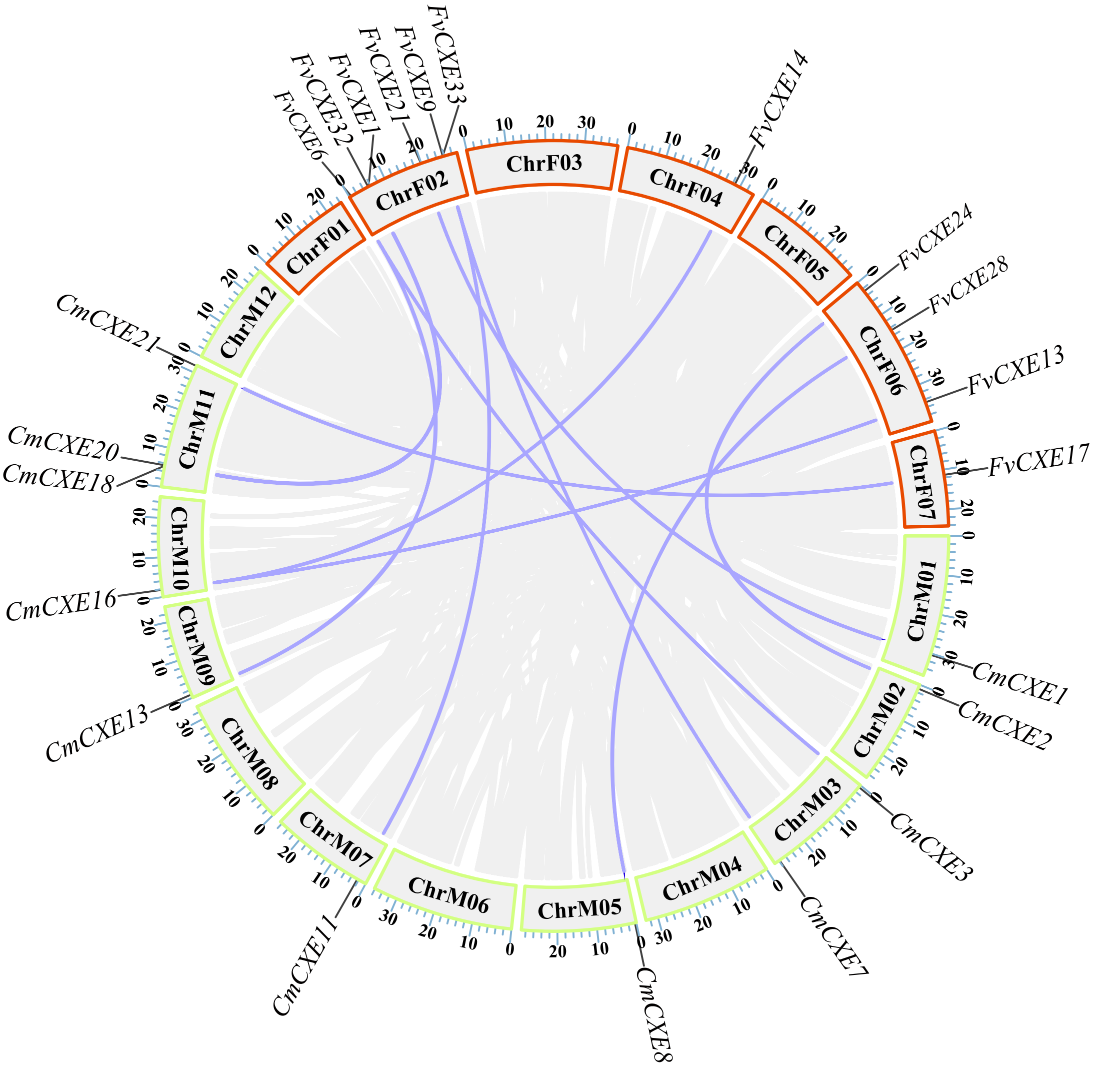

Figure S2. Cross-species syntenic analysis between melon and strawberry CXE genes.

Note: Gray lines represent syntenic blocks between the melon and strawberry genomes, while blue-purple lines highlight syntenic pairs involving CXE genes. This analysis provides evolutionary links between melon CmCXE genes and strawberry *FvCXE* genes, some of which have been functionally validated as aroma-related candidates.

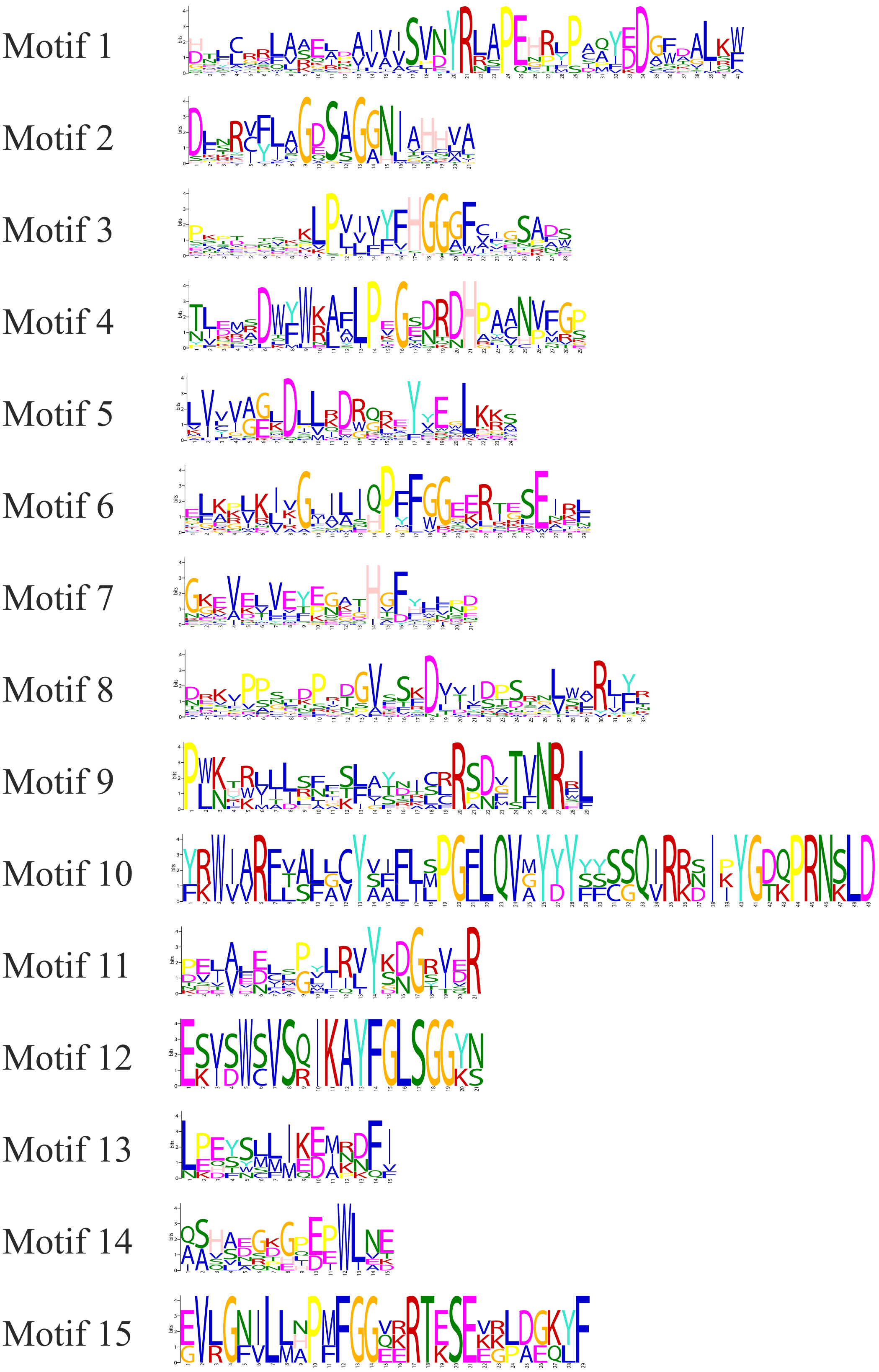

Figure S3. Sequence logos of 15 conserved motifs identified in CmCXE proteins.

Note: The 15 conserved motifs (Motif 1–15) were identified using the MEME suite. The height of each

letter represents the degree of amino acid conservation at that position, with taller letters indicating higher conservation. Different colors represent different amino acid properties. Motif sequences are shown in Figure 3D, and their statistical parameters (E-value, sites, width) are detailed in Table S3.

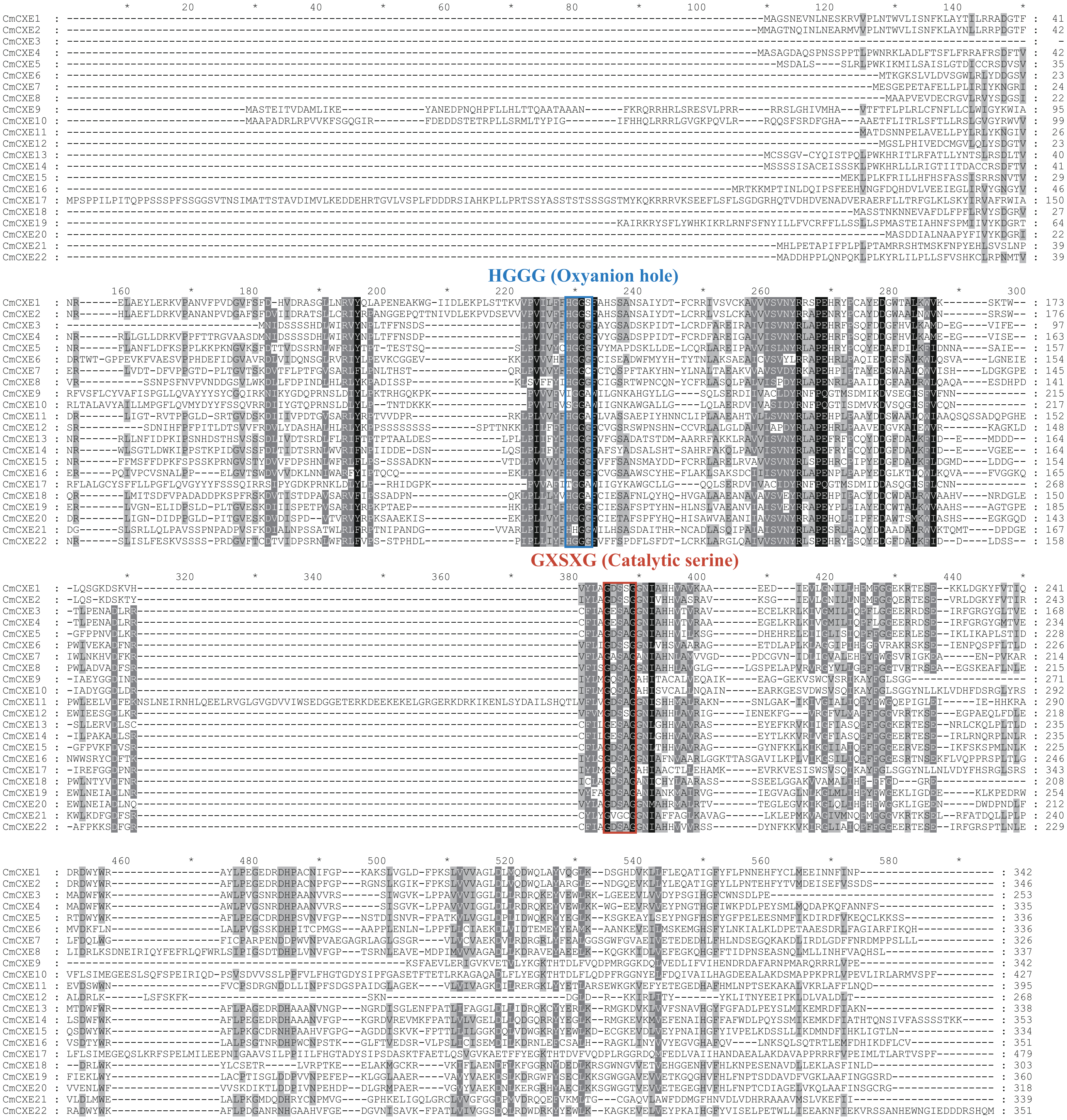

Figure S4. Multiple sequence alignment of the 22 CmCXE proteins generated by GeneDoc.

Note: Conserved and similar residues are shaded in black and gray, respectively. The red box indicates the conserved GXSXG catalytic nucleophile motif (harboring the catalytic serine), and the blue box indicates the conserved HGGG oxyanion hole motif.

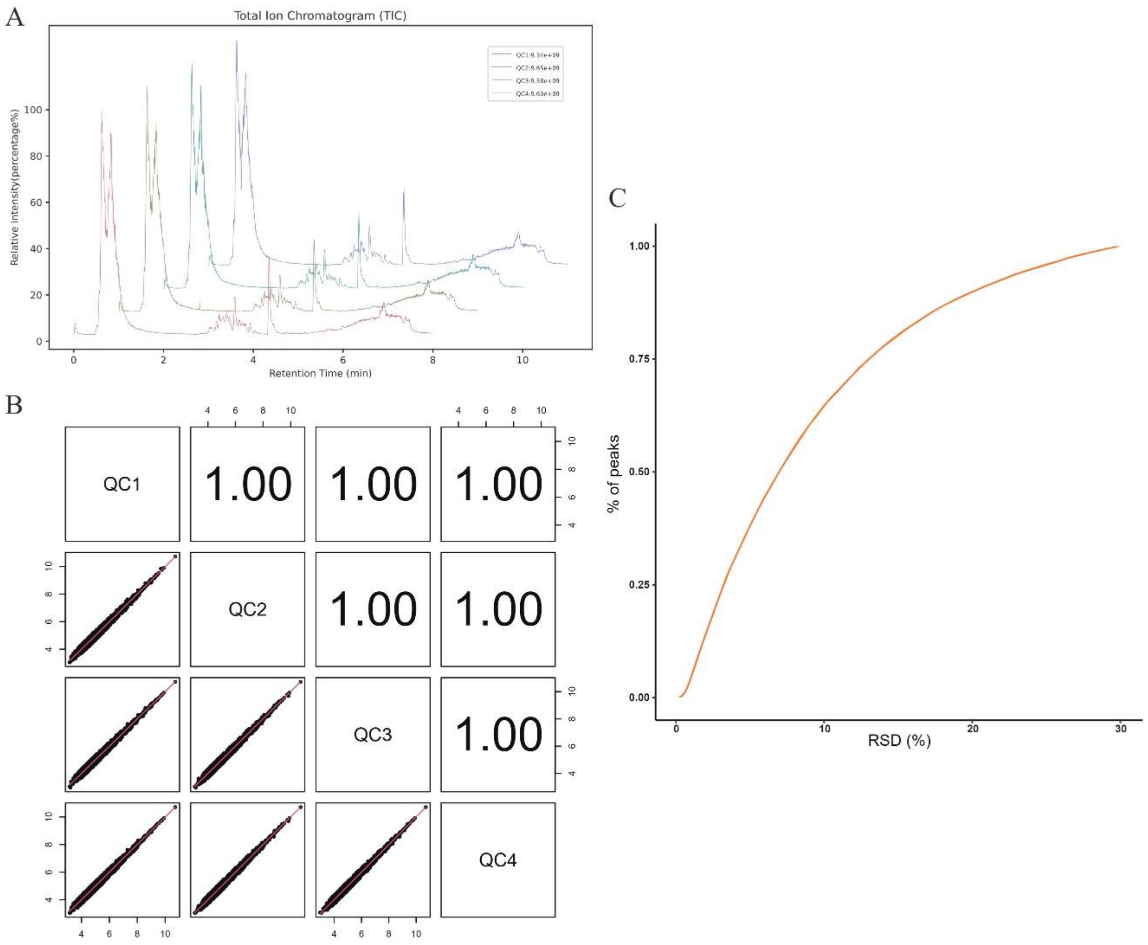

Figure S6. Quality control of the metabolomic profiling. (**A**) Total ion chromatogram (TIC) overlap of all samples in positive and negative ion modes. (**B**) Correlation heatmap of quality-control samples. (**C**) Relative standard deviation (RSD) distribution of all detected metabolites.

Part II: Supplementary Methods (Figure S5 and Tables S11–S21)

Preliminary Functional Characterization of *CmCXE7*

S1. Rationale for Selecting *CmCXE7*

Prior to the genome-wide identification described in the main text, a transcriptomic analysis of postharvest cold storage in thin-skinned melon ('Huaitian No. 1') fruit was conducted under three conditions: (i) 4 °C storage for 7 d followed by 20 °C recovery for 1 d; (ii) 4 °C storage for 8 d; and (iii) freshly harvested fruit (control). This analysis identified 14 aroma-related candidate genes, including the carboxylesterase gene *CmCXE7*. RT-qPCR validation confirmed that *CmCXE7* was significantly upregulated under cold storage and downregulated after recovery at 20 °C, concomitant with the reduction and partial restoration of volatile ester content, respectively. This cold-responsive expression pattern, together with its membership in the carboxylesterase family, motivated its selection for functional validation.

S2. Gene Cloning and Vector Construction

S2.1 Plant Materials and Strains

Fruit at 30 days after pollination from thin-skinned melon (*'Huaitian No. 1'*) was used for RNA extraction. Four-week-old *Nicotiana benthamiana* plants at the four-leaf stage were used for transient expression. *Escherichia coli* DH5α and *Agrobacterium tumefaciens* EHA105 were used for plasmid amplification and plant transformation, respectively.

S2.2 Total RNA Extraction and cDNA Synthesis

Total RNA was extracted from melon mesocarp tissue using the TRIzol method. RNA integrity was verified by 1% agarose gel electrophoresis (Figure S5A), and purity was determined by spectrophotometry. First-strand cDNA was synthesized using the FastKing gDNA Dispelling RT SuperMix kit (Tiangen, Beijing, China). The reaction system and program are listed in Tables S11 and S12.

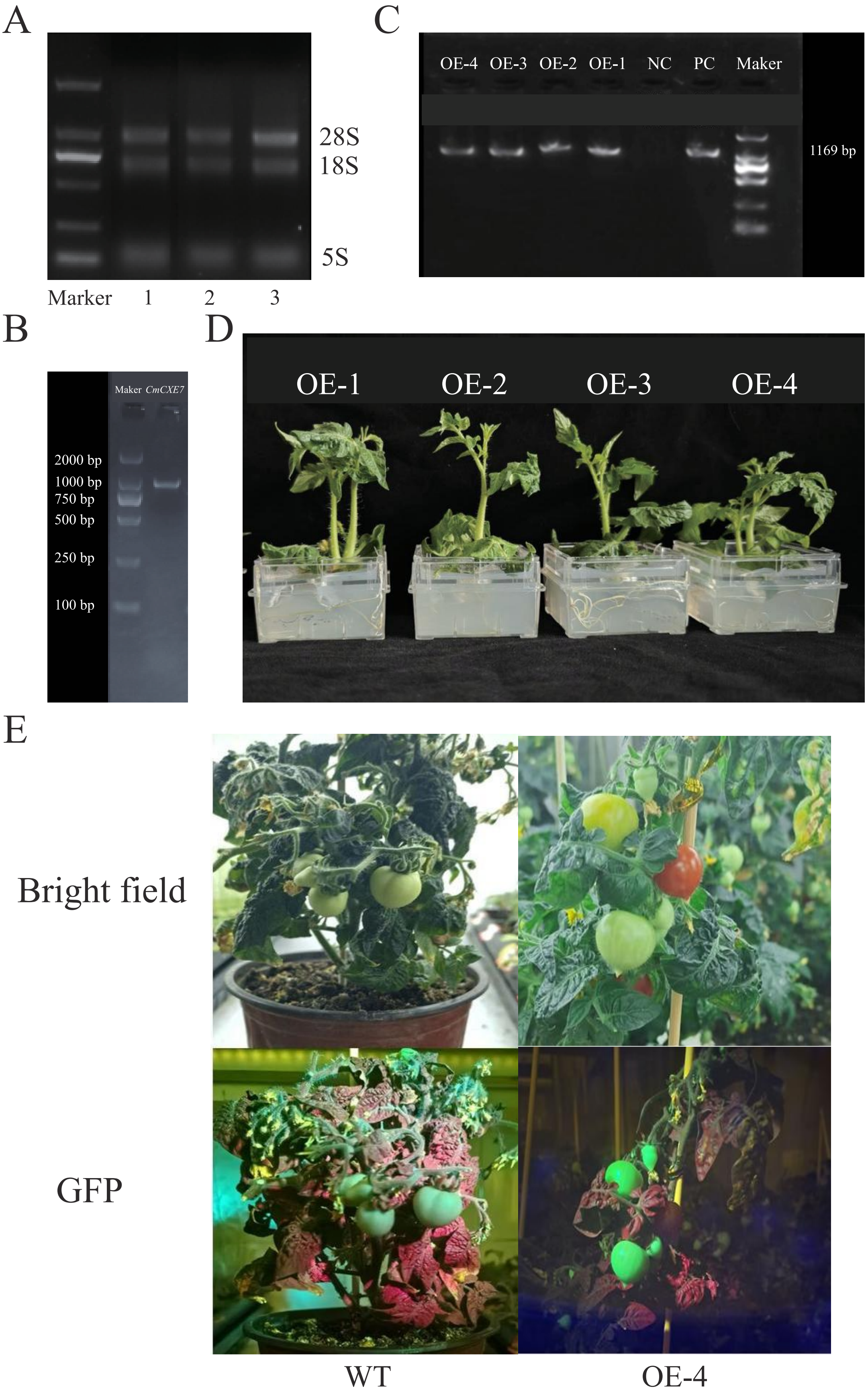

Figure S5: Preliminary functional validation of *CmCXE7*. (**A**)Agarose gel electrophoresis of total RNA from 'Huaitian No. 1' melon fruit under three postharvest treatments: lane 1, 4 °C storage for 7 d followed

by 20 °C recovery for 1 d; lane 2, 4 °C storage for 8 d; lane 3, freshly harvested fruit (control). (**B**) Agarose gel electrophoresis of the *CmCXE7* PCR amplification product. (**C**) PCR detection of transgenic tomato plants (PC: positive control; NC: negative control; OE-1 to OE-4). (**D**) Transgenic tomato seedlings. (**E**) GFP fluorescence observation in transgenic tomato fruits at the green mature stage. WT, wild-type; OE4, *CmCXE7*-overexpressing line.

Table S11. Reverse transcription reaction system.

Component	Volume
5× FastKing-RT SuperMix	4 μL
Total RNA	2 μg
RNase-free ddH_2O	Up to 20 μL

Table S12. Reverse transcription program.

Temperature	Time
42 °C	15 min
95 °C	3 min
4 °C	Hold

S2.3 CmCXE7 Gene Cloning

The full-length coding sequence (CDS, 981 bp) of *CmCXE7* was amplified from melon cDNA using high-fidelity DNA polymerase (KOD-FX, Toyobo, Osaka, Japan). Gene-specific primers were designed with EcoRI and SalI restriction site adapters (Table S13). The PCR amplification system and program are listed in Tables S14 and S15. PCR products were detected by 1.5% agarose gel electrophoresis (Figure S5B) and purified using a gel extraction kit (Axygen, Union City, CA, USA). Sanger sequencing confirmed the identity of the amplicon and revealed two single nucleotide polymorphism (SNP) sites relative to the reference genome sequence.

Table S13. Primers for *CmCXE7* gene cloning.

Primer name	Sequence (5′ → 3′)
35S-*CmCXE7*-EcoRIF	GAGACAGGATCC**GAATTC**ATGGAAT
35S-*CmCXE7*-SalIR	CGGTGACTAGT**GTCGAC**AAGCAGCA

Table S14. PCR amplification system.

Component	Volume
KOD-FX	1 μL
2× PCR buffer	25 μL
dNTP mixture	10 μL
Forward primer	0.2 μM
Reverse primer	0.2 μM
Template cDNA	< 500 ng
ddH_2O	Up to 50 μL

Table S15. PCR amplification program.

Step	Temperature	Time	Cycles
Pre-denaturation	98 °C	2 min	1
Denaturation	98 °C	10 s	
Annealing	60 °C	30 s	34
Extension	68 °C	1 min	
Final extension	68 °C	7 min	1

S2.4 Vector Construction

The purified *CmCXE7* CDS was cloned into the EcoRI/SalI-digested pCAMBIA1300-35S-N-GFP vector using a homologous recombination kit (Thinkgene, Changsha, China), generating a 35S: *CmCXE7*-GFP fusion construct driven by the CaMV 35S promoter. The vector digestion system and homologous recombination system are listed in Tables S16 and S17. The recombinant plasmid was transformed into *E. coli* DH5*α* competent cells and cultured on LB plates containing 50 μg/mL kanamycin at 37 °C for 16 h. Positive clones were verified by colony PCR and Sanger sequencing.

Table S16. Vector digestion system.

Component	Volume (20 μL system)
GFP vector	16 μL
10× FastDigest buffer	2 μL
FastDigest EcoRI	1 μL
FastDigest SalI	1 μL

Table S17. Homologous recombination system.

Component	Volume (10 μL system)
PCR product	4 μL
Digested vector	4 μL
5× TGFast Fusion Mix	2 μL

S3. Subcellular Localization

The 35S:*CmCXE7*-GFP vector was transformed into *A. tumefaciens* EHA105. Transformants were selected on LB plates containing 50 μg/mL kanamycin and 25 μg/mL rifampicin at 28 °C for 2 d. Single colonies were cultured overnight, resuspended in induction medium, and incubated statically at room temperature for 4 h. The cells were then resuspended in infiltration medium to an OD_{600} of 0.5 and infiltrated into the abaxial epidermis of leaves from 4-week-old *N. benthamiana* plants. The formulations of induction and infiltration media are listed in Table S18. At 50 h post-infiltration, GFP fluorescence was observed using a laser scanning confocal microscope (FV1000, Olympus, Tokyo, Japan). The free GFP vector (35S: GFP) was used as a control.

Result: *CmCXE7*-GFP fluorescence was predominantly localized to the plasma membrane, with a weak signal also detected in the nucleus, whereas the free GFP control showed fluorescence throughout the cell (Figure 8 in main text).

Table S18. Formulations of induction and infiltration media.

Medium	Composition
Induction medium	60 mM K_2HPO_4, 33 mM KH_2PO_4, 20 mM sodium citrate, 10 mM $MgSO_4$, 0.2% glucose, 0.2% casamino acids, 50 μg/mL kanamycin, 25 μg/mL rifampicin

Medium	Composition
Infiltration medium	0.5× MS, 10 mM MES, 150 μM acetosyringone, pH 5.6

S4. Generation of Transgenic Tomato Lines

Because the melon genetic transformation system is not yet well established in our laboratory, tomato (*Solanum lycopersicum* cv. MicroTom) was used as a heterologous system for functional validation. The 35S: *CmCXE7*-GFP vector was introduced into tomato via *A. tumefaciens*-mediated transformation. Putative transgenic plants were selected on medium containing 50 mg/L kanamycin.

Genomic DNA was extracted from leaves of putative transgenic plants, and positive lines were identified by PCR using vector-spanning primers that amplify across the promoter–gene junction (Table S19). The expected amplicon size is 1,169 bp. Four independent transgenic lines (OE-1, OE-2, OE-3, OE-4) were confirmed by PCR (Figure S5C) and propagated to the T3 generation (Figure S5D). Among these, the OE-4 line showed the highest transgene expression and was selected for the non-targeted metabolomic analysis described in Section 2.7 of the main text.

To confirm transgene expression at the protein level, GFP fluorescence in T3 transgenic tomato fruits was observed using a LUYOR-3410LB fluorescent protein observation lamp (LUYOR, Shanghai, China) with a 470 nm excitation wavelength. Wild-type (WT) fruits were used as the negative control. Bright-field and GFP fluorescence images were captured under identical exposure settings. Strong green fluorescence was readily detected in OE4 fruits at the green mature stage, whereas the signal became less conspicuous at the red-ripe stage due to the accumulation of lycopene and degradation of chlorophyll, which mask the GFP signal. No GFP fluorescence was observed in WT fruits at either stage (Figure S5E). These results confirm successful expression of the *CmCXE7*-GFP fusion protein in the transgenic line.

Table S19. Vector-spanning primers for transgenic tomato detection.

Primer name	Sequence (5′ → 3′)
TMV-F	TACAACAATTACCACCAACAACA
YFPn-R	TTGTGGCCGTTTACGTCGC

S5. Reagents and Instruments

The main reagents and instruments used in this study are listed in Tables S20 and S21.

Table S20. Main reagents used in this study.

Reagent (catalog no.)	Manufacturer
KOD-FX high-fidelity DNA polymerase (KFX-101)	Toyobo, Osaka, Japan
2× BioRun Magic PCR Mix (#P2011)	BioRun, Wuhan, China
Gel extraction kit	Axygen, Union City, CA, USA
Plasmid miniprep kit	Axygen, Union City, CA, USA
Fast homologous recombination kit (TM0201)	Thinkgene, Changsha, China
DL2000 DNA Marker (3427A)	Takara, Shiga, Japan
TB Green Premix Ex Taq	Takara, Shiga, Japan
FastKing gDNA Dispelling RT SuperMix	Tiangen, Beijing, China
FastDigest EcoRI (FD0274), SalI (FD0644)	Thermo Fisher Scientific, Waltham, MA, USA
DNA Marker 1000 (#RBA00)	BioRun, Wuhan, China
Antibiotics (kanamycin, rifampicin, hygromycin, cefotaxime, carbenicillin)	BioRun, Wuhan, China

Table S21. Main instruments used in this study.

Instrument	Manufacturer
Agarose gel electrophoresis system	Bio-Rad, Hercules, CA, USA
Gel imaging system	Bio-Rad, Hercules, CA, USA
Electric thermostatic water bath (DK-8D)	Yiheng, Shanghai, China
DNA sequencer (ABI 3730)	Applied Biosystems, Foster City, CA, USA
Laser scanning confocal microscope (FV1000)	Olympus, Tokyo, Japan
Gradient PCR instrument (TGreat Expert)	Tiangen, Beijing, China
Fluorescent protein observation lamp (LUYOR-3410LB)	LUYOR, Shanghai, China
