# Supplementary material for: Genome-Wide Identification and Functional Characterization of the Carboxylesterase (CXE) Gene Family in Melon (Cucumis melo L.): CmCXE7 as a Cold-Responsive Regulator of Ester Metabolism
Source: Plants (Basel). 2026 Oct 14;15(20):3144. doi: 10.3390/plants15203144 (PMC13709046; doi:10.3390/plants15203144)
Supplement: Supplementary file 1 [file plants-15-03144-s001.zip › Supplementary Materials/Supplementary Metabolomics Analysis Results/1_QC/1.1_TIC/NEG/TIC.pdf]

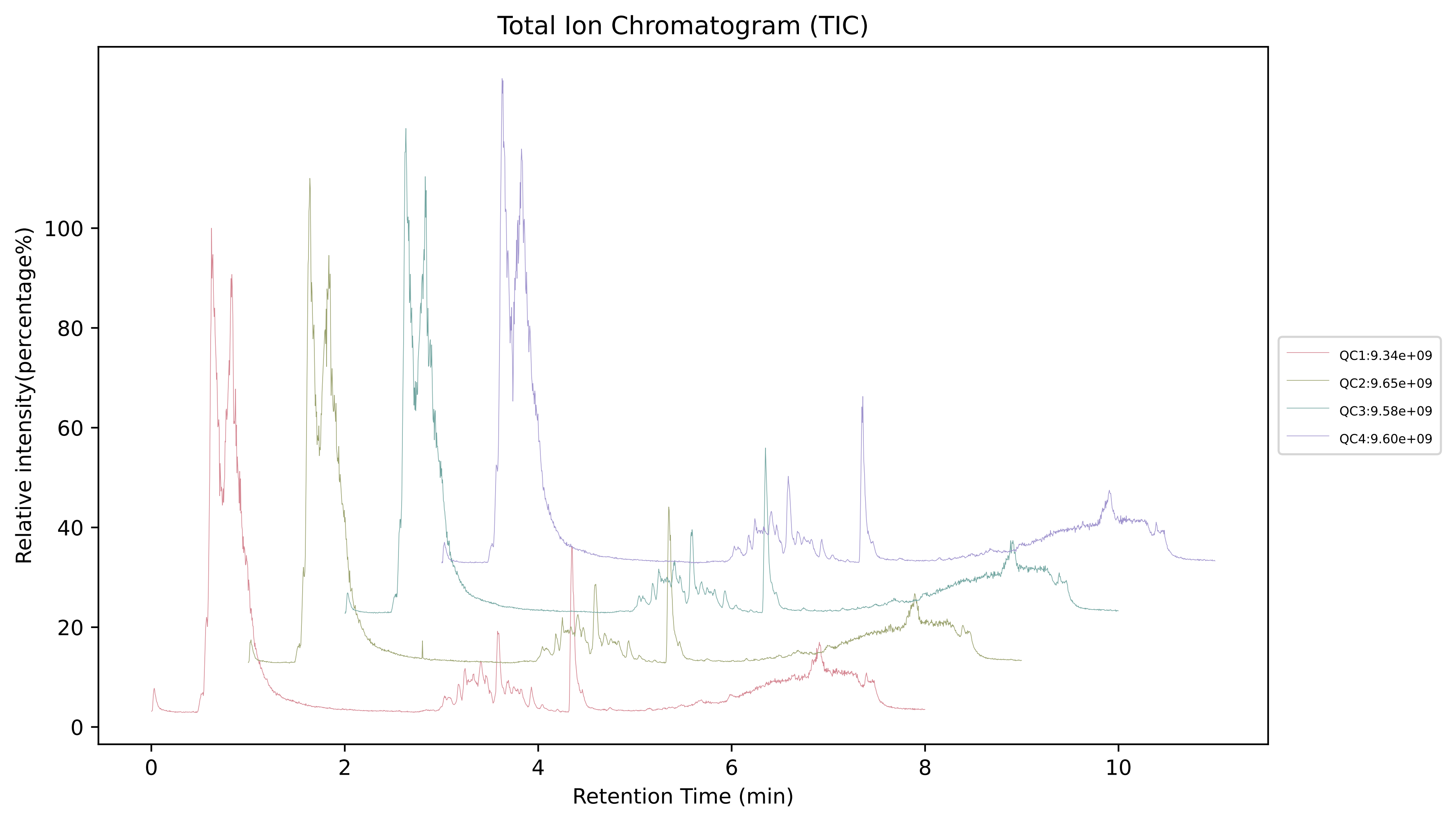
Total Ion Chromatogram (TIC)
Relative intensity(percentage%)
Retention Time (min)
0
20
40
60
80
100
0
2
4
6
8
10
QC1:9.34e+09
QC2:9.65e+09
QC3:9.58e+09
QC4:9.60e+09
