# Supplementary material for: Genome-Wide Identification and Functional Characterization of the Carboxylesterase (CXE) Gene Family in Melon (Cucumis melo L.): CmCXE7 as a Cold-Responsive Regulator of Ester Metabolism
Source: Plants (Basel). 2026 Oct 14;15(20):3144. doi: 10.3390/plants15203144 (PMC13709046; doi:10.3390/plants15203144)
Supplement: Supplementary file 1 [file plants-15-03144-s001.zip › Supplementary Materials/Supplementary Metabolomics Analysis Results/1_QC/1.2_qcCor/POS/QC_Correlation.pdf]

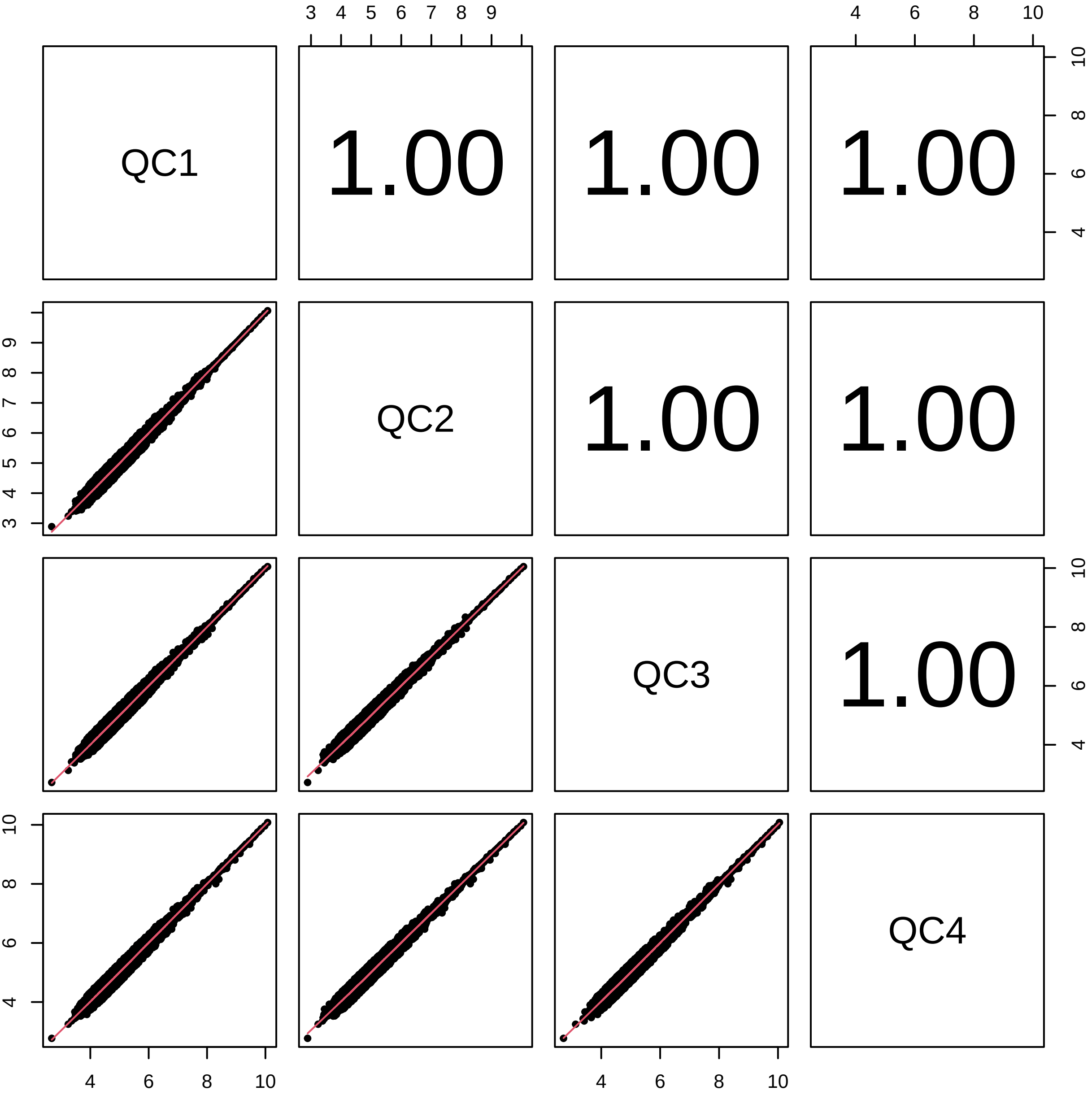
QC1
QC2
QC3
QC4
1.00
1.00
1.00
1.00
1.00
1.00
3 4 5 6 7 8 9
4 6 8 10
