# Supplementary material for: Genome-Wide Identification and Functional Characterization of the Carboxylesterase (CXE) Gene Family in Melon (Cucumis melo L.): CmCXE7 as a Cold-Responsive Regulator of Ester Metabolism
Source: Plants (Basel). 2026 Oct 14;15(20):3144. doi: 10.3390/plants15203144 (PMC13709046; doi:10.3390/plants15203144)
Supplement: Supplementary file 1 [file plants-15-03144-s001.zip › Supplementary Materials/Supplementary Metabolomics Analysis Results/4_Twogroup_diffAnalysis/4.7_heatmap/NEG/heatmap.pdf]

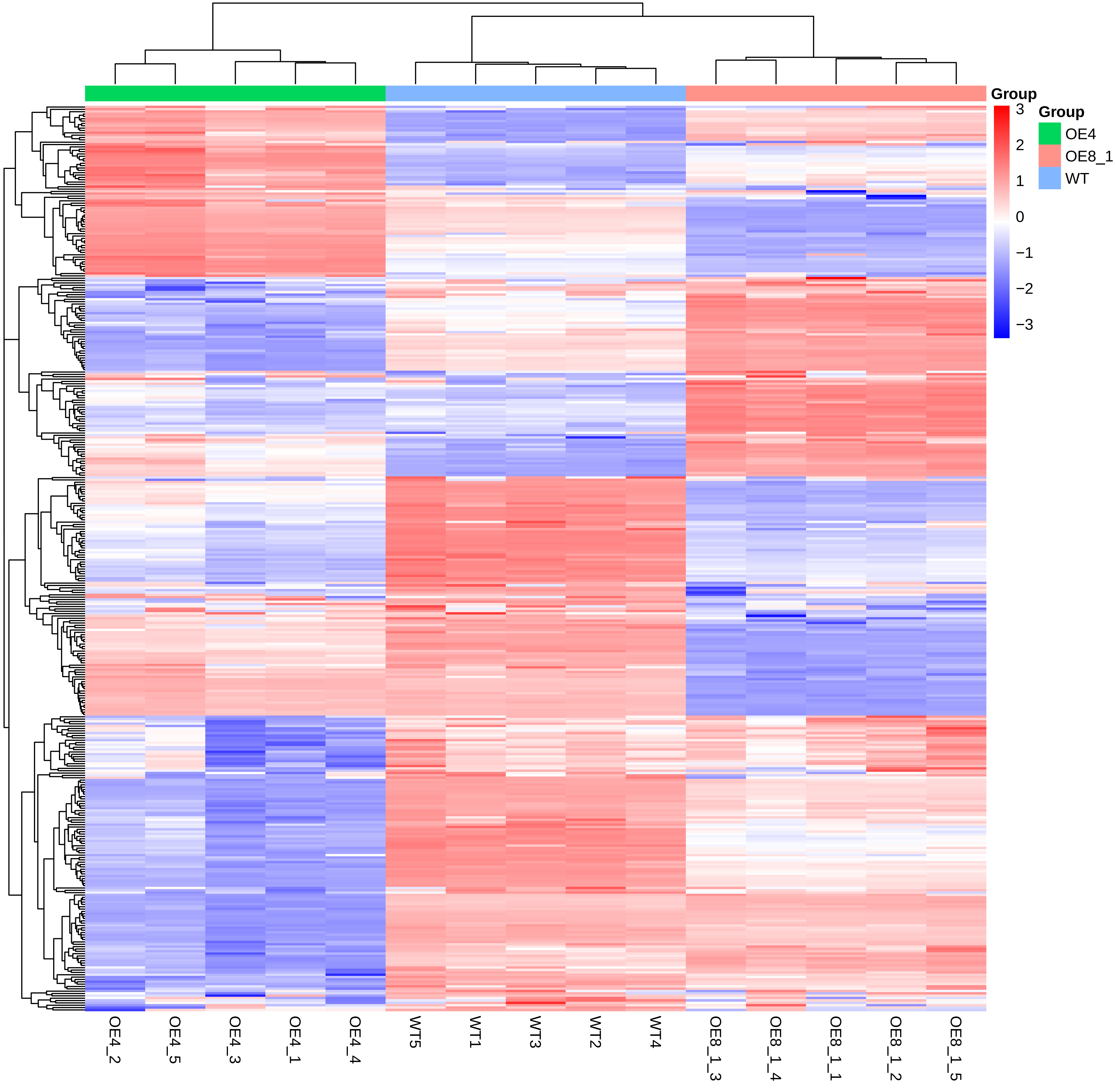

Group
3
2
1
0
−1
−2
−3
Group
OE4
OE8_1
WT
OE4_2
OE4_5
OE4_3
OE4_1
OE4_4
WT5
WT1
WT3
WT2
WT4
OE8_1_3
OE8_1_4
OE8_1_1
OE8_1_2
OE8_1_5
