# Supplementary material for: Genome-Wide Identification and Functional Characterization of the Carboxylesterase (CXE) Gene Family in Melon (Cucumis melo L.): CmCXE7 as a Cold-Responsive Regulator of Ester Metabolism
Source: Plants (Basel). 2026 Oct 14;15(20):3144. doi: 10.3390/plants15203144 (PMC13709046; doi:10.3390/plants15203144)
Supplement: Supplementary file 1 [file plants-15-03144-s001.zip › Supplementary Materials/Supplementary Metabolomics Analysis Results/4_Twogroup_diffAnalysis/4.8_trend/Merge/cluster_plots.pdf]

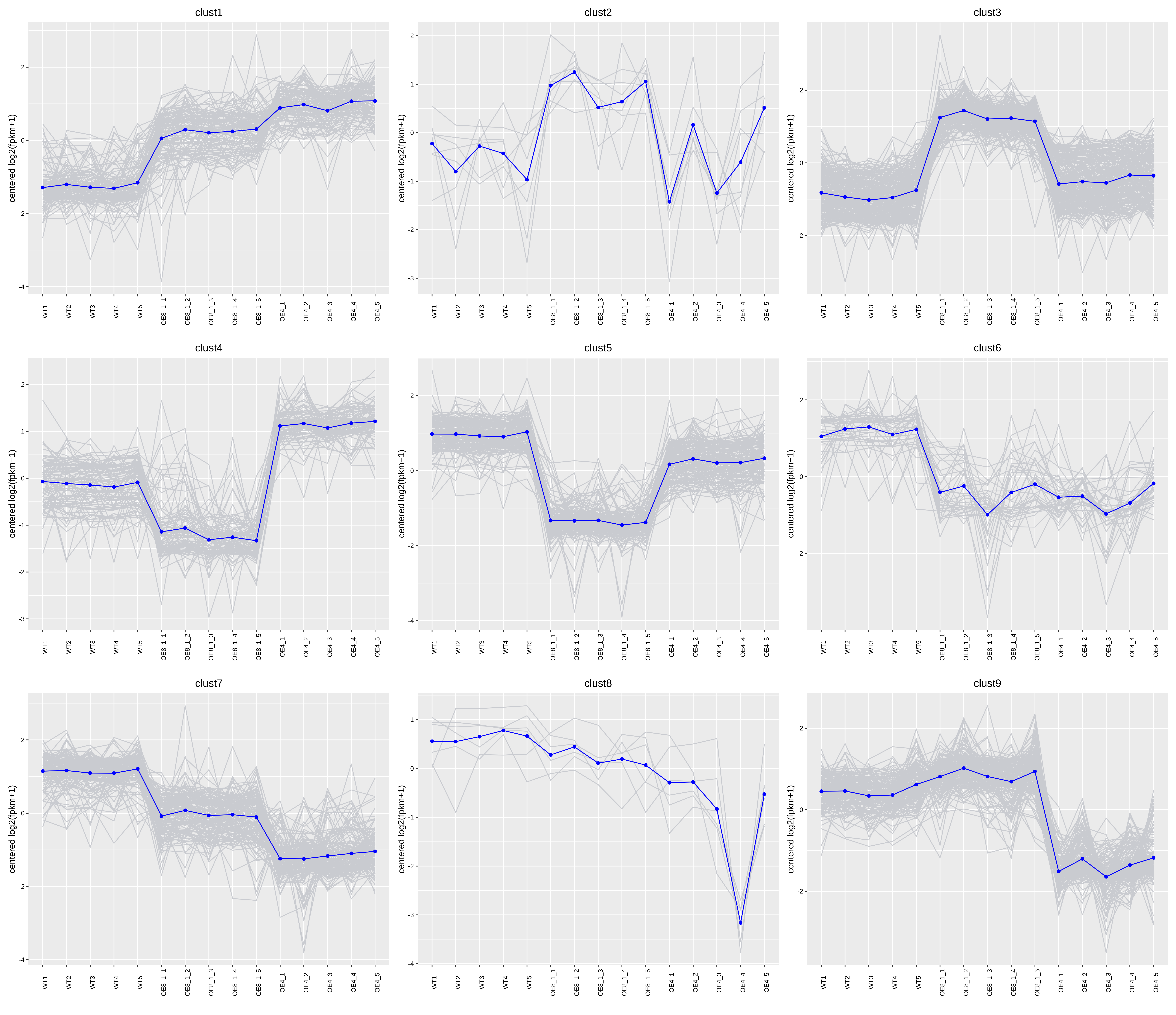
clust1
clust2
clust3
clust4
clust5
clust6
clust7
clust8
clust9
centered log2(fpkm+1)
WT1
WT2
WT3
WT4
WT5
OE8_1_1
OE8_1_2
OE8_1_3
OE8_1_4
OE8_1_5
OE4_1
OE4_2
OE4_3
OE4_4
OE4_5
