# Supplementary material for: Genome-Wide Identification and Functional Characterization of the Carboxylesterase (CXE) Gene Family in Melon (Cucumis melo L.): CmCXE7 as a Cold-Responsive Regulator of Ester Metabolism
Source: Plants (Basel). 2026 Oct 14;15(20):3144. doi: 10.3390/plants15203144 (PMC13709046; doi:10.3390/plants15203144)
Supplement: Supplementary file 1 [file plants-15-03144-s001.zip › Supplementary Materials/Supplementary Metabolomics Analysis Results/4_Twogroup_diffAnalysis/4.9_correlation/Merge/WT_vs_OE4/cor_DEM.pdf]

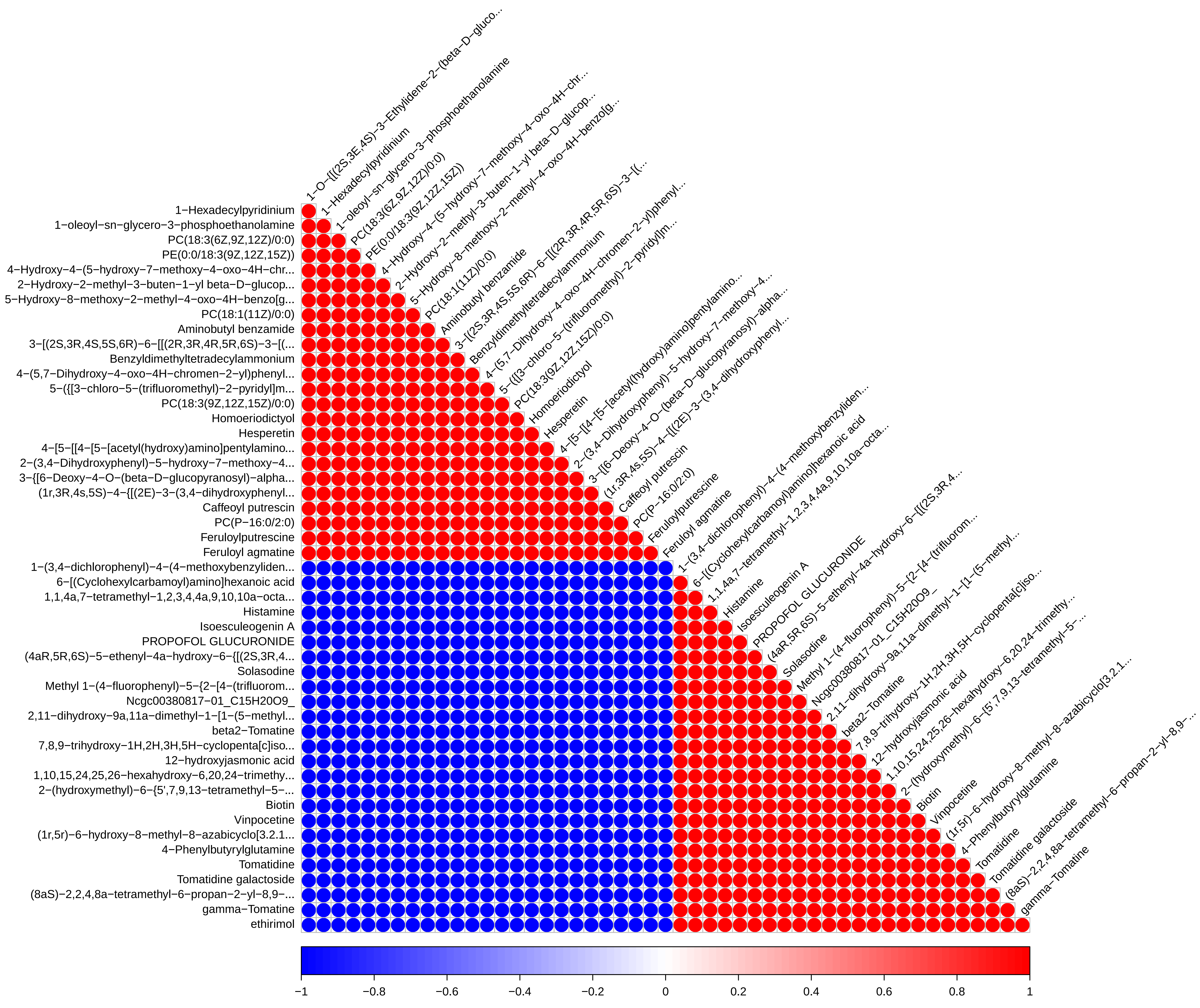
1−O−{[(2S,3E,4S)−3−Ethylidene−2−(beta−D−gluco...
1−Hexadecylpyridinium
1−oleoyl−sn−glycero−3−phosphoethanolamine
PC(18:3(6Z,9Z,12Z)/0:0)
PE(0:0/18:3(9Z,12Z,15Z))
4−Hydroxy−4−(5−hydroxy−7−methoxy−4−oxo−4H−chr...
2−Hydroxy−2−methyl−3−buten−1−yl beta−D−glucop...
5−Hydroxy−8−methoxy−2−methyl−4−oxo−4H−benzo[g...
PC(18:1(11Z)/0:0)
Aminobutyl benzamide
3−[(2S,3R,4S,5S,6R)−6−[[(2R,3R,4R,5R,6S)−3−[(...
Benzyldimethyltetradecylammonium
4−(5,7−Dihydroxy−4−oxo−4H−chromen−2−yl)phenyl...
5−({[3−chloro−5−(trifluoromethyl)−2−pyridyl]m...
PC(18:3(9Z,12Z,15Z)/0:0)
Homoeriodictyol
Hesperetin
4−[5−[[4−[5−[acetyl(hydroxy)amino]pentylamino...
2−(3,4−Dihydroxyphenyl)−5−hydroxy−7−methoxy−4...
3−{[6−Deoxy−4−O−(beta−D−glucopyranosyl)−alpha...
(1r,3R,4s,5S)−4−{[(2E)−3−(3,4−dihydroxyphenyl...
Caffeoyl putrescin
PC(P−16:0/2:0)
Feruloylputrescine
Feruloyl agmatine
1−(3,4−dichlorophenyl)−4−(4−methoxybenzyliden...
6−[(Cyclohexylcarbamoyl)amino]hexanoic acid
1,1,4a,7−tetramethyl−1,2,3,4,4a,9,10,10a−octa...
Histamine
Isoesculeogenin A
PROPOFOL GLUCURONIDE
(4aR,5R,6S)−5−ethenyl−4a−hydroxy−6−{[(2S,3R,4...
Solasodine
Methyl 1−(4−fluorophenyl)−5−{2−[4−(trifluorom...
Ncgc00380817−01_C15H20O9_
2,11−dihydroxy−9a,11a−dimethyl−1−[1−(5−methyl...
beta2−Tomatine
7,8,9−trihydroxy−1H,2H,3H,5H−cyclopenta[c]iso...
12−hydroxyjasmonic acid
1,10,15,24,25,26−hexahydroxy−6,20,24−trimethy...
2−(hydroxymethyl)−6−{5',7,9,13−tetramethyl−5−...
Biotin
Vinpocetine
(1r,5r)−6−hydroxy−8−methyl−8−azabicyclo[3.2.1...
4−Phenylbutyrylglutamine
Tomatidine
Tomatidine galactoside
(8aS)−2,2,4,8a−tetramethyl−6−propan−2−yl−8,9−...
gamma−Tomatine
ethirimol
−1
−0.8
−0.6
−0.4
−0.2
0
0.2
0.4
0.6
0.8
1
