# Supplementary material for: Genome-Wide Identification and Functional Characterization of the Carboxylesterase (CXE) Gene Family in Melon (Cucumis melo L.): CmCXE7 as a Cold-Responsive Regulator of Ester Metabolism
Source: Plants (Basel). 2026 Oct 14;15(20):3144. doi: 10.3390/plants15203144 (PMC13709046; doi:10.3390/plants15203144)
Supplement: Supplementary file 1 [file plants-15-03144-s001.zip › Supplementary Materials/Supplementary Metabolomics Analysis Results/4_Twogroup_diffAnalysis/4.9_correlation/NEG/WT_vs_OE4/cor_DEM.pdf]

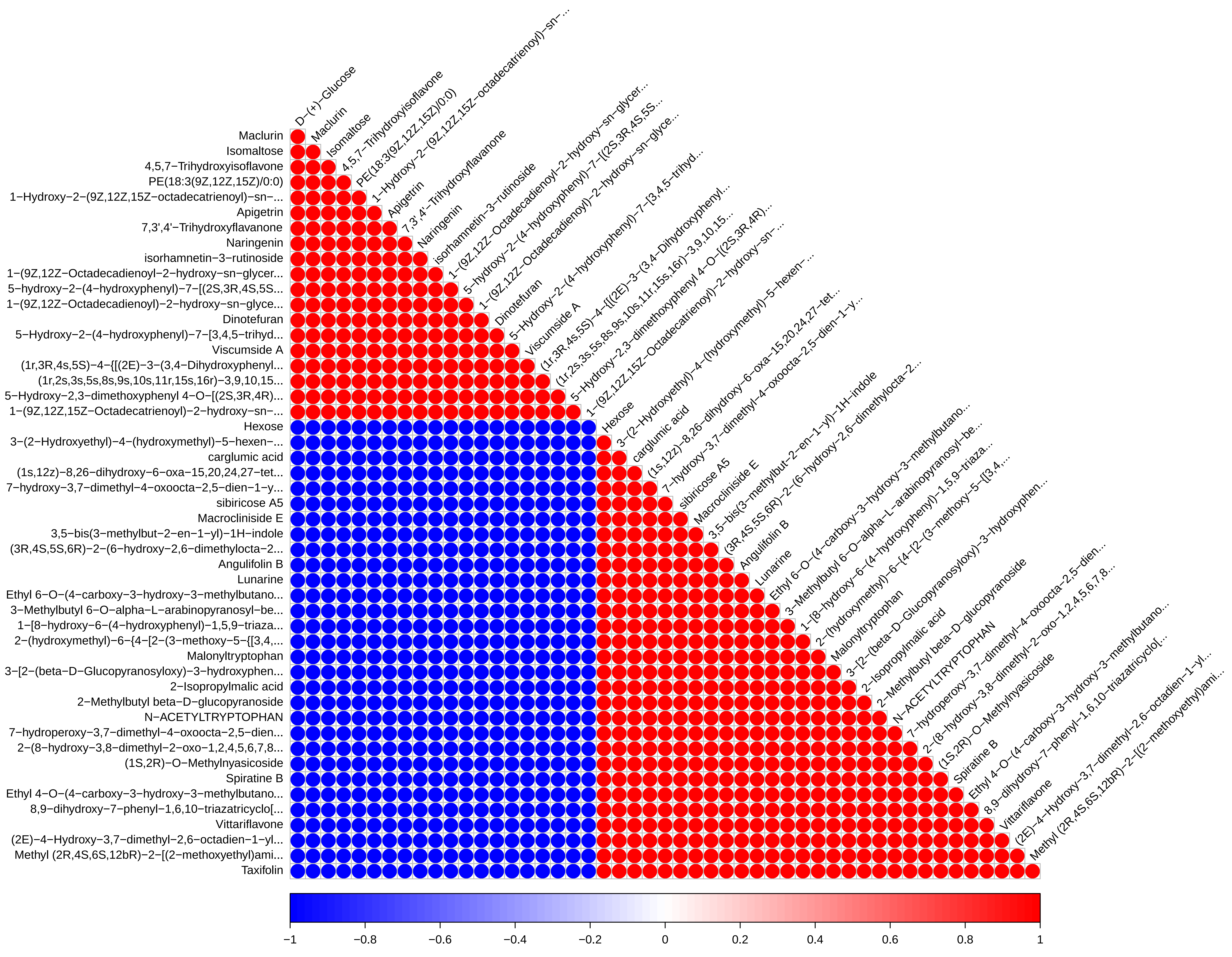

D−(+)−Glucose
Maclurin
Isomaltose
4,5,7−Trihydroxyisoflavone
PE(18:3(9Z,12Z,15Z)/0:0)
1−Hydroxy−2−(9Z,12Z,15Z−octadecatrienoyl)−sn−...
Apigetrin
7,3',4'−Trihydroxyflavanone
Naringenin
isorhamnetin−3−rutinoside
1−(9Z,12Z−Octadecadienoyl−2−hydroxy−sn−glycer...
5−hydroxy−2−(4−hydroxyphenyl)−7−[(2S,3R,4S,5S...
1−(9Z,12Z−Octadecadienoyl)−2−hydroxy−sn−glyce...
Dinotefuran
5−Hydroxy−2−(4−hydroxyphenyl)−7−[3,4,5−trihyd...
Viscumside A
(1r,3R,4s,5S)−4−{[(2E)−3−(3,4−Dihydroxyphenyl...
(1r,2s,3s,5s,8s,9s,10s,11r,15s,16r)−3,9,10,15...
5−Hydroxy−2,3−dimethoxyphenyl 4−O−[(2S,3R,4R)...
1−(9Z,12Z,15Z−Octadecatrienoyl)−2−hydroxy−sn−...
Hexose
3−(2−Hydroxyethyl)−4−(hydroxymethyl)−5−hexen−...
carglumic acid
(1s,12z)−8,26−dihydroxy−6−oxa−15,20,24,27−tet...
7−hydroxy−3,7−dimethyl−4−oxoocta−2,5−dien−1−y...
sibiricose A5
Macrocliniside E
3,5−bis(3−methylbut−2−en−1−yl)−1H−indole
(3R,4S,5S,6R)−2−(6−hydroxy−2,6−dimethylocta−2...
Angulifolin B
Lunarine
Ethyl 6−O−(4−carboxy−3−hydroxy−3−methylbutano...
3−Methylbutyl 6−O−alpha−L−arabinopyranosyl−be...
1−[8−hydroxy−6−(4−hydroxyphenyl)−1,5,9−triaza...
2−(hydroxymethyl)−6−{4−[2−(3−methoxy−5−{[3,4,...
Malonyltryptophan
3−[2−(beta−D−Glucopyranosyloxy)−3−hydroxyphen...
2−Isopropylmalic acid
2−Methylbutyl beta−D−glucopyranoside
N−ACETYLTRYPTOPHAN
7−hydroperoxy−3,7−dimethyl−4−oxoocta−2,5−dien...
2−(8−hydroxy−3,8−dimethyl−2−oxo−1,2,4,5,6,7,8...
(1S,2R)−O−Methylnyasicoside
Spiratine B
Ethyl 4−O−(4−carboxy−3−hydroxy−3−methylbutano...
8,9−dihydroxy−7−phenyl−1,6,10−triazatricyclo[...
Vittariflavone
(2E)−4−Hydroxy−3,7−dimethyl−2,6−octadien−1−yl...
Methyl (2R,4S,6S,12bR)−2−[(2−methoxyethyl)ami...
Taxifolin
−1
−0.8
−0.6
−0.4
−0.2
0
0.2
0.4
0.6
0.8
1
